# Diet-induced ketosis in adult patients with subacute acquired brain injury: a feasibility study

**DOI:** 10.3389/fmed.2023.1305888

**Published:** 2024-03-19

**Authors:** Maria G. P. Edwards, Jens R. Andersen, Derek J. Curtis, Christian G. Riberholt, Ingrid Poulsen

**Affiliations:** ^1^Department of Nutrition, Exercise and Sports, University of Copenhagen, Copenhagen, Denmark; ^2^Department of Brain and Spinal Cord Injury, Neuroscience Centre, Copenhagen University Hospital – Rigshospitalet, Copenhagen, Denmark

**Keywords:** ketogenic diet (KD), ketosis, **β**-hydroxybutyrate (BHB), medium-chain triglycerides (MCT), acquired brain injury (ABI), subarachnoid haemorrhage (SAH), stroke, traumatic brain injury (TBI)

## Abstract

**Background:**

Research in animal models on cerebral metabolism after brain injury highlights the potential benefits of ketosis in reducing secondary brain injury, but studies in humans are lacking.

**Aim:**

This study aimed to examine if a 6-week ketogenic diet intervention with added medium-chain triglycerides (MCT) was feasible in adult patients with acquired brain injury in the subacute phase, whether ketosis could be achieved and maintained, and to what extent serious adverse reactions, adverse reactions, serious adverse events, and adverse events occured.

**Methods:**

Patients ≥18 years of age diagnosed with subacute acquired brain injury and an expectation of hospitalisation ≥6 weeks were included in the intervention group. Patients not included in the intervention group were included in a standard care reference group. The intervention consisted of a ketogenic diet supplemented with MCT to obtain a plasma concentration of β-hydroxybutyrate (BHB) ≥0.5 mmol/L. Patients who were enterally fed were given KetoCal® 2.5:1 LQ MCT Multi Fiber (Nutricia A/S, Allerød, Denmark), supplemented with Liquigen® (Nutricia A/S, Allerød, Denmark). Patients consuming oral nutrition were given KetoCal® 2.5:1 LQ MCT Multi Fiber supplemented with Liquigen®, in addition to ketogenic meals.

**Results:**

During a 13-week inclusion period, 12 of 13 eligible patients (92% [95% CI: 67% to 99%]) were included in the intervention group, and 17 of 18 excluded patients (94% [95% CI: 74% to 99%]) were included in the reference group. Eight patients (67%) completed the 6-week intervention. It took a median of 1 day to achieve ketosis from starting a 100% MCT ketogenic diet, and it was maintained for 97% of the intervention period after ketosis was obtained. There were no serious adverse reactions to the MCT ketogenic diet, and patients experienced adverse reactions not considered serious in 9.5% of days with the intervention. The MCT ketogenic diet was accepted by patients on all intervention days, and in the two patients transitioning from enteral feeding to oral intake, there were no complications related to transitioning.

**Conclusion:**

Intervention with MCT ketogenic diet is feasible and tolerated for 6 weeks in hospitalised adult patients with subacute acquired brain injury. Randomised controlled trials are needed to assess the benefits and harms of the MCT ketogenic diet and the effect on patients’ recovery.

**Clinical trial registration:** ClinicalTrials.gov, identifier [NCT04308577].

## Introduction

Acquired brain injury (ABI) is a frequent cause of injury-related morbidity and mortality worldwide. Global statistics for the incidence of ABI are lacking; however, globally, 27 million new cases of traumatic brain injury (TBI) were reported in 2016. The incidence of stroke was 12 million in 2019 when it was the third greatest cause of disability-adjusted life years globally in all ages and the second for people over 50 years old (1–3). The primary injury resulting immediately from ABI leads to secondary injury, which occurs gradually and includes an entire cascade of cellular, chemical, tissue, or blood vessel changes in the brain that contribute to further destruction of brain tissue (4). To improve clinical outcomes and rehabilitation, attempts to limit the secondary injury cascade are important. The duration of the secondary injury cascade and its effects on, e.g., metabolic cellular dysfunction and inflammation are not yet fully known.

Under normal physiological conditions, the brain’s energy supply is mainly covered by glucose. The brain can utilise ketone bodies (KB), such as β-hydroxybutyrate (BHB) and acetoacetate (AcAc), as alternative cerebral energy substrates, supplying up to 60% of the basal cerebral energy requirements (5–7). In addition to its activity as an energetic metabolite, BHB is also a signalling metabolite that affects epigenetic gene regulation and cellular function and has important neuroprotective effects (8, 9). In ABI, glucose deficiency occurs as a result of a reduced rate of cerebral glucose metabolism and a decreased availability of cerebral extracellular glucose.

Animal studies have shown that increasing KB metabolism through fasting or diet-induced ketosis promotes brain resistance to stress and injury and attenuates acute cerebral injury (10, 11). In the liver, β-oxidation of medium-chain triglycerides (MCT) (C6–C10) is a rapid process, making MCT a suitable ketogenic substrate (12, 13). The MCT ketogenic diet (MCT-KD) has been shown to be a non-pharmacological treatment in refractory epilepsy, where beneficial metabolic changes have an effect on seizure control (13, 14). The mechanisms of the ketogenic diet (KD) are now investigated in several other disorders, e.g., Alzheimer’s disease, cancer, diabetes, and mental illness (13–15). Research with the KD in ABI is still in its early stages. In acute ABI, five human studies investigating cerebral ketone metabolism have been published, showing that ketosis can be safely achieved (9, 16–19).

Ten patients with acute TBI and ventriculostomy catheter to monitor intracranial pressure were included in the study by Arora et al. (18). The primary feasibility outcome was the achievement of ketosis, and the secondary outcomes were adverse effects related to the KD. Enrolled patients were given intravenous (i.v.) fluids free of glucose in the emergency room, operating room, or intensive care unit (ICU). Ketogenic formulation (KetoVie 4:1®, Cambrooke) with additional protein supplementation was initiated on average within 23 h of admission to the ICU. Eight out of 10 patients achieved ketosis, blood glucose (BG) decreased in most patients, two patients developed hypertriglyceridaemia, one patient developed hypoglycaemia, and no other adverse effects were noted. Arora et al. (18) concludes that a KD is feasible in the management of acute TBI patients.

White et al. (19) investigated feasibility and safety by administrating ketogenic enteral nutrition for 6 days (KetoCal®, Nutricia) to 20 mechanically ventilated critically ill patients with acute ABI, included within 48 h of admission to ICU. The primary endpoint was to investigate the possibility of inducing ketosis using ketogenic enteral nutrition, measured as BHB and AcAc concentration in plasma and cerebrospinal fluid (CSF). Secondary endpoints were to determine the effect of a KD on intracranial pressure, cerebral perfusion pressure, and metabolic and acid–base parameters. Patients were continuously monitored for any adverse effects. White et al. (19) conclude that enteral ketogenic nutrition is well tolerated in patients with acute severe ABI and can be safely administered.

Bernini et al. (9) used cerebral microdialysis to measure levels of BHB and AcAc interstitially in the brain of 34 patients with acute TBI, measured in a fasted state, and by half and full enteral nutrition, either with standard enteral nutrition (Promote Fibres Plus®, Abbott Nutrition—24 patients) or with enteral nutrition supplemented with MCT (MCT-enriched Peptamen AF®, Nestlé—10 patients). Both products had a carbohydrate content of 13.5–14 g/100 mL and a MCT content of 6.5 g/1,000 kcal and 23 g/1,000 kcal, respectively. They found that KB levels in the blood correlated with levels in the brain and that the onset of nutrition was associated with a significant gradual decrease in KB levels both in the blood and in the brain. Nutrition with added MCT did not increase KB in the blood but did significantly increase plasma-free C8 and C10, as well as a significant increase in C8 and C10 in the brain. Levels of KB interstitially in the brain during the fasting acute phase of TBI were associated with increasing age, and Bernini et al. (9) suggest that there are other determinants affecting KB in the brain in addition to nutritional status.

Ritter et al. (16) randomised 20 patients with acute TBI to either enteral nutrition without carbohydrate and with 175 g fat/L (intervention: nine patients) or enteral nutrition with 141 g carbohydrate/L and 36.8 g fat/L (control: 11 patients). In the control group, the BG level increased whilst remaining unchanged at the fasting level in the intervention group. The intervention group had lower blood lactate concentrations and higher concentrations of KB in the blood. The urine-nitrogen balance was better in the intervention group, receiving a higher protein intake. CSF lactate concentration and cerebral lactate production were not significantly different in the two groups. Ritter et al. (16) conclude that a carbohydrate-free diet may have benefits for patients with acute TBI, by meeting energy and protein requirements without causing hyperglycaemia.

Robertson et al. (17) randomised 21 patients in coma after acute TBI, and patients received either glucose i.v. or NaCl (0.45%) i.v. for 5 days. All patients received 3% amino acids (75 g/day), and the control group received 5% glucose (106 g/day). The intervention group got no calories besides protein. There was no difference in blood or CSF glucose concentration in the two groups, but the control group had higher plasma insulin levels. The intervention group had higher blood levels of BHB, AcAc, pyruvate, glycerol, and free fatty acids than the control group. The cerebral oxygen turnover was similar in the two groups, but in the control group, glucose was the only energy substrate for the brain. In the intervention group, BHB and AcAc replaced glucose in 16% of the brain’s total energy turnover. CSF lactate concentration and cerebral lactate production were lower in the intervention group. Robertson et al. (17) conclude that early-stage glucose administration after acute TBI suppresses ketogenesis, increases lactate production, and limits the damaged brain’s supply of non-glycolytic energy substrates.

No clinical studies investigating the KD in subacute ABI have been published, although there is a solid basis for a hypothesis of a beneficial effect in these patients based on the experimental results. Therefore, we designed a study to investigate if MCT-KD is feasible in adult patients with subacute ABI during a 6-week intervention. The primary aims were to examine to what extent it was possible to recruit patients, attain and maintain ketosis, the occurrence of serious adverse reactions (SARs) and adverse reactions (ARs) not considered serious to the MCT-KD, the acceptance of treatment, and transition from enteral to oral intake. Our exploratory aims were changes in functional ability from admission to 4 weeks and differences in serious adverse events (SAEs) and adverse events (AEs) not considered serious in the MCT-KD group and in a standard care reference (SCR) group.

## Materials and methods

This single-site feasibility study was conducted over 5 months on patients with subacute ABI admitted to the Department of Brain and Spinal Cord Injury, Neuroscience Centre, Copenhagen University Hospital, Rigshospitalet, Denmark. This study is reported following the Consolidated Standards of Reporting Trials (CONSORT) statement (20). Ethics approval was granted by the Capital Region’s Committee on Health Research Ethics (H-20018775), and the study was registered at clinicaltrials.gov (Identifier: NCT04308577) on 3rd June 2020. The study was conducted in accordance with the principles of the Declaration of Helsinki (21); written informed consent was obtained from the patients or the patient’s next of kin in the case of patients without the capacity to consent. The protocol was approved by the Danish Data Protection Agency.

### Participants

Patients ≥18 years diagnosed with subacute ABI (TBI, subarachnoid haemorrhage (SAH), anoxic brain injury, or stroke), an expectation of hospitalisation ≥6 weeks in a department for highly specialised neurorehabilitation, were included. Exclusion criteria from 29 September 2020 to 16 December 2020 were if KD was contraindicated (22) (Supplementary Table 1), if patients had diabetes mellitus, were in statin therapy for hypercholesterolaemia, if the treating physician assessed the patient not to be eligible due to atherosclerosis, and at >10% weight loss during hospitalisation after ABI, before admission to the department. On 17 December 2020, a protocol amendment with exclusion criteria adjustments was approved by Capital Region’s Committee on Health Research Ethics. The adjustments were made after gaining further scientific and clinical knowledge about which patients to exclude. From 17 December 2020 to 9 February 2021, patients were excluded if KD was contraindicated (22) (Supplementary Table 1), diabetes mellitus was dysregulated, or was treated with medication for elevated triglycerides. Excluded patients and patients who did not consent to participate in the study were asked for consent to participate in a SCR group, with the primary purpose of investigating the safety of the MCT-KD and also providing the possibility of generating data for potential future power calculations. Following study entry, the patients were weaned off standard nutrition and MCT-KD was increased over 1 to 2 days. Patients were fed in three ways: by nasogastric tube in bolus, via a percutaneous endoscopic gastrostomy as a continuous infusion over a few hours per meal, or orally. Energy requirements were calculated from the Harris-Benedict equation corrected for stress and activity factor (23). All nutrition and fluids were registered daily and calculated using a Danish internet-based software ‘Vitakost’, for nutritional contents (24). If deficiencies occurred, they were supplemented accordingly.

### Nutritional intervention

Patients in the intervention group received an enteral ketogenic formulation that also can be consumed orally—KetoCal® 2.5:1 LQ MCT Multi Fiber (KetoCal® 2.5:1) (Nutricia A/S, Allerød, Denmark), supplemented with additional MCT–Liquigen® (Nutricia A/S, Allerød, Denmark). Patients capable of oral intake received KetoCal® 2.5:1 and Liquigen® orally, complemented with ketogenic meals produced by Hvidovre Hospital diet kitchen. The KetoCal® 2.5:1 ratio product consists of 82.9% fat, 2.9% carbohydrate, 1.4% dietary fiber, 11.8% protein, and 3.6 g MCT per 100 mL. Liquigen® is a fat emulsion consisting of approximately 50% MCT and 50% water. Per 50 g fat (100 mL), there is 0.34 g C6 (caproic acid), 28.9 g C8 (caprylic acid), 19.3 g C10 (capric acid), and 0.095 g C12 (lauric acid). Thixo-D Cal-Free® (Ecogreen Technologies, Buntingford, England), a xanthan gum thickener, was used to thicken the consistency of drinks and foods for patients with dysphagia. A wide selection of low-carb/ketogenic food products were used as substitutes for standard food products, such as ketogenic desserts, baking products, snacks, and candy, during occupational therapy and when requested. Patients on oral nutrition were allowed ketogenic meals *ad libitum*.

### Feasibility outcomes

The primary objective of this trial was to assess feasibility. The intervention was considered to be feasible if all of the criteria in Table 1 were attained. Limits to determine feasibility were estimated from the results of previous trials (18, 19, 25–27).

**Table 1:** Feasibility outcomes.

Outcomes	Measures	Limit to be feasible
Recruitment of patients	Percent of included patients to eligible patients	≥ 60% of eligible patients included (25)
Attaining ketosis*	Days to attain ketosis* from initiating 100% MCT ketogenic diet	≥ 75% of patients attaining ketosis* within 5 days from initiating 100% MCT ketogenic diet (18, 19)
Maintaining ketosis*	Percent of days in ketosis* since ketosis* was first obtained	≥ 75% of days in ketosis* since ketosis* was first obtained (26)
Occurrence of serious adverse reactions and adverse reactions not considered serious to MCT ketogenic diet	Percent of intervention days with serious adverse reactions and adverse reactions	≤ 5% of intervention days with serious adverse reactions in 100% of patients ≤ 30% of intervention days with adverse reactions in ≥75% of patients (18, 19)
Acceptance of treatment	Yes/No, percent of intervention days	≥ 75% of patients accepting the treatment in ≥75% of intervention days (26)
Transition from enteral to oral intake	Percent of intervention days with complications when transitioning from enteral to oral intake	≤ 30% of intervention days with complications in ≥75% of patients transitioning from enteral to oral intake (27)

*Ketosis was defined as a mean value of the three-daily blood β-hydroxybutyrate measurements ≥ 0.5 mmol/L. MCT, medium-chain triglycerides.

#### Attaining ketosis

To assess the level of ketosis and fluctuations in BG, blood BHB (b-BHB) and BG were monitored three times daily during the study: morning, mid-day, and evening. Measurements were predominantly taken postprandial (within 4 h after a meal) and not in a fasted state. This was because the first meal was given at 6 am, and meals were then given every 3 to 4 h until the evening. b-BHB and BG were sampled by a finger pricker and FreeStyle Precision Neo® (Abbott Laboratories A/S, Copenhagen, Denmark), a BG device that measures both b-BHB and BG with two different test strips. A day in ketosis was defined as a mean value of the three-daily b-BHB measurements ≥0.5 mmol/L.

#### Occurrence of serious adverse reactions and adverse reactions to MCT ketogenic diet

Serious adverse events (SAEs), adverse events (AEs), serious adverse reactions (SARs), adverse reactions (ARs), and suspected unexpected serious adverse reactions (SUSARs) were monitored during the trial and reported according to ICH E6 Good Clinical Practice guideline (28). SAEs were defined as an adverse event that results in death, is life-threatening, requires hospitalisation or prolongation of existing hospitalisation, results in persistent or significant disability or incapacity, or requires intervention to prevent permanent impairment or damage, whether considered related to the trial intervention or not. AEs were defined as any untoward medical occurrence in a patient that does not necessarily have a causal relationship with the intervention. SARs and ARs were defined as any harmful and undesirable reaction with a direct causal relationship to the intervention, serious or not considered serious (28). SAEs, AEs, SARs, and ARs were assessed and documented daily in the MCT-KD group. In the SCR group, SAEs and AEs were assessed and documented weekly.

##### Biochemical analysis for safety

Venous plasma blood samples were analysed weekly (triglycerides, potassium, sodium, magnesium, creatinine and phosphate) or every 2 weeks (alanine aminotransferase (ALAT), alkaline phosphate, bilirubin and International Normalised Ratio (INR)) in the MCT-KD group to assess safety. Haemoglobin A1c (HbA1c) was analysed at inclusion and completion/exclusion. Supplementary blood samples were analysed as needed for clinical reasons. All blood samples were taken postprandial and not in a fasted state, as the first meal was at 6 am, and blood samples were taken between 7 am and 8 am. Results from blood samples taken as part of standard care in the SCR group were not registered in this study.

#### Acceptance of treatment and transition from enteral to oral intake

Acceptance of treatment and complications during the transition from enteral to oral intake was assessed and documented daily. A day with diarrhoea was defined as thin defecation more than three times per day, and constipation was defined as more than 2 days without defecation, where a physician or nurse had noted in the patient’s medical record that the patient was constipated. A day with nausea, vomiting, stomachache, or headache was defined as one episode noted in the patient’s medical record or reported by the nursing staff to the investigator. Acceptance of treatment was defined as patients accepting the enteral feeding and/or the ketogenic meals given and willingly consuming as much of the nutrition as they could. For incapable patients, accepting treatment meant that the next of kin accepted the treatment and that the patient consumed an acceptable amount of the nutrition given. An acceptable amount was defined as nutrition enough to keep the patient’s body weight stable. Patients or their next of kin (for patients without the capacity to consent) were asked daily if they wanted to continue in the study. Complications during the transition from enteral to oral intake were defined as any complications assessed by occupational therapists to be related to the transition to oral intake, e.g., aspiration due to dysphagia.

### Exploratory outcomes

Registration form for adults, ‘Danish Head Trauma Database - quality development of treatment and rehabilitation 2015-2017’, was used to assess patients’ recovery stage by several functional measurements. In this study, the following measurements were used in both the MCT-KD and SCR groups at admission and after 4 weeks for all patients: Glasgow Coma Scale (GCS) (29), Early Functional Abilities (EFA) (30), Functional Independence Measure (FIM) (31), and Functional Oral Intake Scale (FOIS) (32). The Ranchos Los Amigos Scale (RLAS) (33) was only used for TBI patients.

#### Bioelectrical impedance analysis and weighing

Patients in the MCT-KD group were weighed and measured with bioelectrical impedance analysis—InBody S10® (InBody, Denmark) at inclusion, weekly, and completion/exclusion. Bedridden patients were weighed on a digital scale attached to a ceiling hoist or chair scale, and mobile patients were weighed on a digital bathroom scale. Body weight measured as part of standard care in the SCR group was not registered in this study.

### Statistical analysis

We aimed in this study to include 12 patients. As this is the first feasibility study in patients with subacute ABI, we followed the recommendations of Julious (34), as a formal power calculation was not feasible. For the feasibility outcomes, we compared the lower limits of the confidence interval with our predefined levels, resembling a one-sided t-test. No formal statistical comparison was carried out due to the low sample size. All data were presented as median and interquartile range as data were not normally distributed.

## Results

### Feasibility outcomes

#### Recruitment of patients

**Figure 1:**
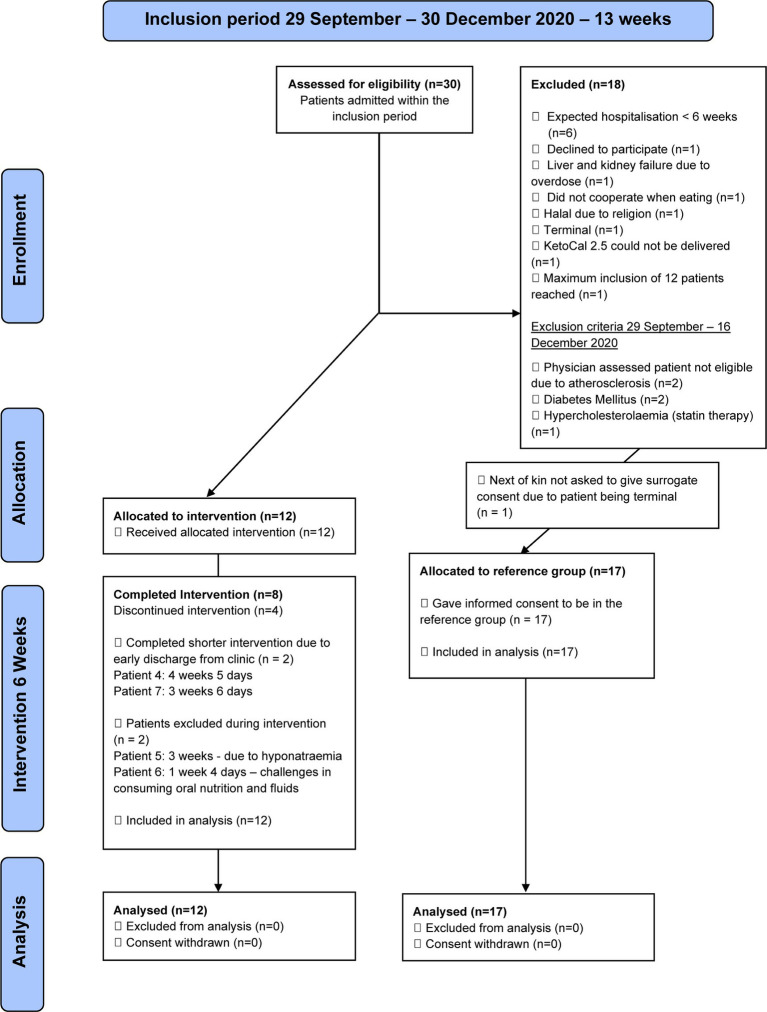
Flow diagram describing the inclusion and exclusion of patients in the study.

It was possible to include 12 of 13 (92% [95% CI: 67% to 99%]) eligible patients (40% of all patients admitted to the department) during a 13-week inclusion period. Only one patient declined to participate, but 17 patients were excluded for other reasons (Figure 1). Surrogate consent was obtained on behalf of all patients without the capacity to consent. Out of the excluded patients, 17 of 18 patients (94% [95% CI: 74% to 99%]) consented to be allocated to a SCR group. The one patient not included in the SCR group was terminal.

Eight patients (67% [95% CI: 39% to 86%]) completed the 6-week intervention. Two patients were excluded during the intervention by the clinically responsible physician; one patient after 1 week and 4 days, due to a loss of appetite and challenges in consuming oral nutrition and fluid independently. The other patient was excluded after 3 weeks due to dangerously low plasma sodium. Two patients completed a shorter intervention due to early discharge from the hospital. One after 4 weeks and 5 days and the other after 3 weeks and 6 days (Figure 1).

##### Baseline characteristics

Characteristics of the patients included in the MCT-KD group and SCR group are summarised in Table 2. Two patients in the MCT-KD group and one patient in the SCR group were diagnosed with hydrocephalus at the ICU weeks before the intervention. During the intervention, it was discovered that one patient had had a cholecystectomy which resulted in bile acid diarrhoea for several weeks before the intervention. The diarrhoea stopped when treated with Colestyramin®, after 18 days of bile acid diarrhoea during the intervention. One patient had constipation as an adverse effect of the medication Frisium®, which was treated with laxatives regularly and resulted in diarrhoea and constipation for several weeks before the intervention. The patient who was excluded after 1 week and 4 days had a loss of appetite since the ABI and had challenges in consuming oral nutrition and fluid independently for several weeks before the intervention. Patients’ comorbidities and previous diagnoses are listed in Supplementary Table 2.

**Table 2:** Patients’ baseline characteristics.

Variable	MCT - Ketogenic diet group (*n* = 12)	Standard care reference group (*n* = 17)
**Age (years)**	60 (56 to 71)	59 (52 to 66)
Traumatic brain injury	60 (60 to 66) (*n* = 3)	33 (33 to 34) (*n* = 2)
Anoxic brain injury	-	56 (49 to 62) (*n* = 2)
Subarachnoid haemorrhage	60 (53 to 66) (*n* = 3)	52 (51 to 56) (*n* = 3)
Stroke	61 (53 to 69) (*n* = 6)	60 (57 to 68) (*n* = 10)
Gender (Male/Female)	8/4	12/5
Body mass index	21.6 (19.6 to 23.9)	25.5 (22.9 to 28.8)
**Diagnosis**		
Traumatic brain injury	3	2
Anoxic brain injury	-	2
Subarachnoid haemorrhage	3	3
Stroke	6	10
Time of injury prior to admission to Department of Brain Injury (days)	48 (37 to 70)	28 (25 to 40)
Time of injury prior to MCT-KD intervention (days)	52 (44 to 77)	na
Glasgow Coma Scale (unsedated) within 48 h of injury	8 (3 to 11)	6 (3 to 9) (*n* = 16)
**At admission to Department of Brain Injury**		
Glasgow Coma Scale	12 (12 to 14)	13 (13 to 14)
Early Functional Abilities	44 (42 to 51) (*n* = 8)	59 (43 to 66) (*n* = 15)
Functional Independence Measure	20 (18 to 27) (*n* = 10)	23 (21 to 30) (*n* = 15)
Functional Oral Intake Scale	1 (1 to 3) (*n* = 11)	3 (1 to 4) (*n* = 14)
Ranchos Los Amigos Scale (for Traumatic brain injury)*	5 (4 to 6) (*n* = 3)	5 (4 to 5) (*n* = 2)
Tracheostomy (Yes/No)	4/8	2/15
**Nutrition**		
Enteral nutrition	10	13
Nasogastric tube	6	7
Percutaneous endoscopic gastrostomy	4**	6
Orally	2	4

Data are presented as number of patients or median (IQR).*Ranchos Los Amigos Scale was measured in all traumatic brain injury patients.**One of the patients with percutaneous endoscopic gastrostomy also consumed nutrition orally.n, number; na, not applicable; MCT-KD, ketogenic diet with added medium-chain triglycerides.

#### Attaining ketosis

**Figure 2:**
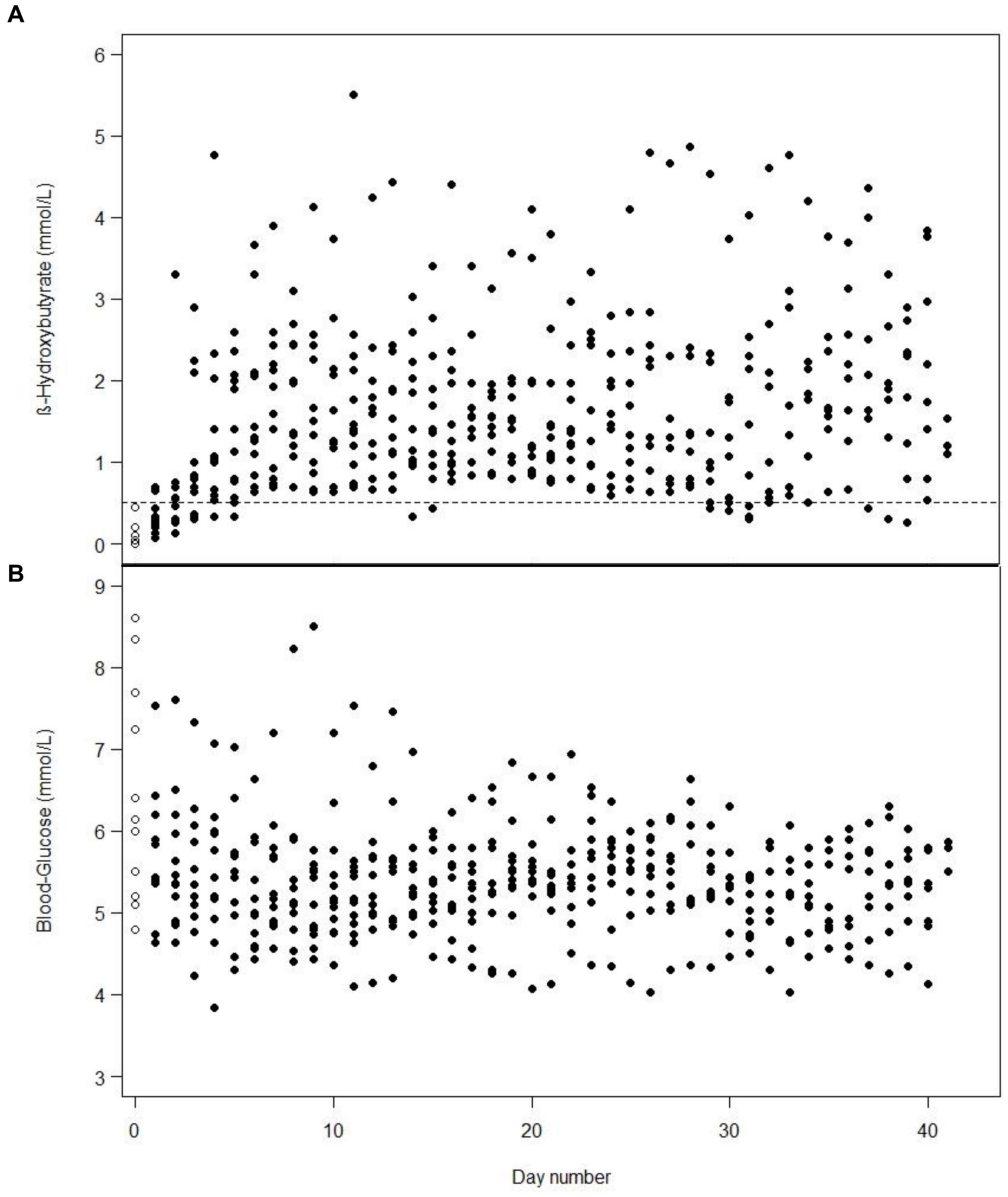
One dot is the mean value of the three-daily blood β-hydroxybutyrate **(A)** or blood glucose **(B)** measurements in one patient from day 1 of intervention, e.g., includes measurements from the first days when the patients were weaning off standard nutrition. White dots with a black border are baseline measurements on day 1. On some days, the values for several patients are identical and, therefore, overlap.

It took a median of 1 day (min 0 to max 3 days) to achieve ketosis from starting 100% MCT-KD, and it was maintained for 97% of the time after ketosis was attained. One patient was already in ketosis the day before 100% MCT-KD was initiated, on day 2 of weaning off standard enteral nutrition, due to the decreasing amount of carbohydrates and a gradual increase of KetoCal® 2.5:1. Mean b-BHB increased, and mean BG decreased for all patients over 2 weeks before stabilising for 4 weeks. The daily mean b-BHB (A) and BG (B) values per patient from intervention day 1 are shown in Figure 2. Total number of days in and out of ketosis for each patient is found in Supplementary Figure 1. Diagrams of all daily b-BHB and BG measurements throughout the intervention per patient are found in Supplementary Figures 2–13. Out of the planned three-daily b-BHB and BG measurements, 5% were missed.

#### Occurrence of serious adverse events and reactions, and adverse events and reactions

Eight of 12 (67% [95% CI: 39% to 86%]) patients in the MCT-KD group and 14 of 17 (82% [95% CI: 59% to 94%]) in the SCR group experienced at least one SAE. Three of 12 (25% [95% CI: 9% to 53%]) patients in the MCT-KD group and 1 of 17 (6% [95% CI: 1% to 27%]) in the SCR group experienced at least one AE not considered serious (Table 3). The number of SAEs and AEs not considered serious per patient were 1.1 and 0.25 in the MCT-KD group and 1.2 and 0.06 in the SCR group, respectively. There were no observed SARs or SUSARs in the MCT-KD group, and 9.5% of days with ARs not considered serious (Table 4). The ARs not considered serious had no effect on the acceptance of treatment.

**Table 4:** Number and percent of days with clinical adverse reactions out of 428 intervention days and number of patients experiencing at least one biochemical adverse reaction.

Clinical adverse reaction	Number of days with clinical adverse reactions out of total 428 intervention days	Biochemical adverse reactions	Patients experiencing at least one biochemical adverse reaction
Nausea Vomit Diarrhoea Constipation Stomachache Headache	13 (3%) 9 (2%) 14 (3.3%) 1 (0.2%) 2 (0.5%) 2 (0.5%)	Hypertriglyceridaemia Hyponatraemia Hypoglycaemia	8 (67%) 4 (33%) 0 (0%)

Hypertriglyceridaemia > 2.0 mmol/L, hyponatraemia < 135 mmol/L, hypoglycaemia < 3.5 mmol/L.

**Table 3:** Adverse events and reactions during the 6-week intervention period.

	Ketogenic diet group (*n* = 12)	Standard care reference group (*n* = 17)
**Patients experiencing at least one**		
Adverse events - *n* (%)	3 (25)	1 (6)
Serious adverse events - *n* (%)	8 (67)	14 (82)
Adverse reactions - *n* (%)	9 (75)	na
Serious adverse reactions - *n* (%)	*-*	na
SUSAR - *n* (%)	*-*	na
**Total number of events**		
Adverse events (*n* = 4) - *n* (%)	3 (75)	1 (25)
Serious adverse events (*n* = 33) - *n* (%)	13 (39)	20 (61)
Adverse reactions (*n* = 53) - *n* (%)	53 (100)	na
Serious adverse reactions (*n* = 0) - *n* (%)	-	na
SUSAR (*n* = 0) - *n* (%)	-	na

n, number; na, not applicable; SUSAR, Suspected unexpected serious adverse reaction.

##### Biochemical analysis for safety

There were no changes in potassium, magnesium, creatinine, phosphate, ALAT, alkaline phosphate, bilirubin, or INR that could be associated with the MCT-KD. However, there were examples of skewed blood values in patients who had previously elevated liver values due to alcohol overconsumption or medication. HbA1c decreased in 11 patients (92%) and normalised in patients with elevated values. In eight patients (67%), plasma triglycerides measured postprandial were above the reference value of 2.0 mmol/L in over half of the measurements. The highest concentration of plasma triglycerides (>10.0 mmol/L) was observed after 23 days in a patient diagnosed with diabetes mellitus type 2 during the intervention, and the patient was treated with Metformin® from day 23. The patient’s plasma triglycerides were elevated at inclusion (4.10 mmol/L). Total fat and MCT intake was lowered until the patient’s plasma triglycerides decreased to 5.58 mmol/L on day 41. Weekly plasma triglyceride measurements for all patients from baseline to end of week 6 are found in Supplementary Figure 14. One patient had hyponatraemia (<135 mmol/L) (35, 36) before the intervention, which normalised when increasing sodium chloride supplementation but recurred several times during the intervention despite sodium chloride supplements. Three patients developed hyponatraemia during the intervention. Out of the three, one patient was excluded due to severe hyponatraemia (122 mmol/L) on day 21. On two occasions, it was discovered that the patient did not take the sodium chloride supplements prescribed.

#### Acceptance of treatment and transitioning from enteral feeding to oral intake

The MCT-KD was accepted in 100% of intervention days by all patients. Patients receiving enteral nutrition (*n* = 10) were weaned off standard enteral feeding products and gradually increased in KetoCal® 2.5:1 within 1 to 2 days. Patients receiving solely oral nutrition (*n* = 2) received ketogenic meals in addition to KetoCal® 2.5:1 and started on 100% MCT-KD on day 1. Liquigen® was added at mean day 9 from starting 100% MCT-KD. The timing of when Liquigen® was added was dependent on how the patient tolerated the MCT-KD, and whether there were any ARs not considered serious. The starting dosage of Liquigen® was 30–50 mL per day. The maximum dosage given was 100 mL Liquigen® per day, given to a patient with diabetes mellitus type 2 to try to achieve higher ketosis. The remaining patients received a dosage between 50 and 90 mL per day. Total MCT intake consisted of both MCT from KetoCal® 2.5:1 and Liquigen® and can be seen in Table 5. Three patients were receiving nutrition orally before the intervention, and two patients started receiving nutrition orally during the intervention. The two patients who transitioned from enteral feeding to oral intake during the intervention did not experience any complications related to transitioning to oral intake. The rest of the patients (*n* = 7) were fully enterally fed during the intervention. Patients receiving ketogenic meals had a higher carbohydrate and protein intake and a lower total MCT intake due to less KetoCal® 2.5:1 (Table 5). The percent difference between baseline nutrition estimates and 100% MCT-KD shows an increase in energy, fat, MCT, and dietary fiber intake, and a lowering of carbohydrate, sugars, and protein (Table 6).

**Table 5:** Mean daily nutrition intake for all patients on 100% ketogenic diet with added medium-chain triglycerides (Liquigen®) from day 1 to the end of the intervention.

	Mean daily nutrient consumption during the intervention													
Patient	Energy (kcal)	Fat (g)	MCT total (g)	C6 (mg)	C8 (mg)	C10 (mg)	C12 (mg)	Carbohydrate (g)	Sugars (g)	Dietary Fiber (g)	Protein (g)	Fat E%	Carbohydrate E%	Protein E%
**1**	2,194	209.6	71.6	245.7	42,784.4	28,546.4	210.4	14.0	10.2	13.9	56.9	82.4	3.7	10.2
**2**	3,118	282.4	56.9	182.1	33,873.9	22,599.9	182.7	29.1	19.6	19.9	101.2	81.2	5.0	13.1
**3**	2,305	218.5	68.6	195.4	40,604.6	27,088.1	197.8	15.2	11.1	15.2	62.3	83.9	4.0	11.0
**4**	2,282	216.1	60.6	176.3	35,893.0	23,945.3	187.5	15.2	11.0	15.2	62.1	83.8	4.0	11.1
**5**	2,775	249.4	44.7	111.8	26,311.1	17,551.1	151.7	26.8	17.7	18.5	99.8	79.4	5.2	14.7
**6**	2,134	190.5	32.0	72.6	18,740.0	12,500.0	110.4	22.3	13.8	13.7	78.7	78.9	5.5	14.9
**7**	1,971	187.8	60.6	185.0	35,980.5	24,004.5	196.8	12.8	9.3	12.8	52.6	84.1	3.9	10.9
**8**	2,289	216.2	62.9	149.8	36,941.5	24,641.5	201.0	15.6	11.4	15.4	63.2	83.6	4.1	11.2
**9**	2,456	228.5	65.2	220.5	38,916.7	25,965.5	196.5	21.2	14.8	15.4	72.8	82.4	4.7	11.9
**10**	2,343	223.3	74.7	245.3	44,569.5	28,858.3	211.3	15.0	10.9	15.0	61.5	84.3	3.9	10.7
**11**	2,277	209.9	57.7	239.1	34,897.1	23,287.9	171.1	18.3	12.0	13.9	74.7	81.6	4.4	13.3
**12**	1,975	187.2	58.4	163.9	34,553.5	22,660.8	180.4	13.1	9.5	13.1	53.6	83.7	4.0	11.1
**Mean**	**2,343**	**218.3**	**59.5**	**182.3**	**35,338.8**	**23,470.8**	**183.1**	**18.2**	**12.6**	**15.2**	**69.9**	**82.4**	**4.4**	**12.0**

Patient 2, 5, 6, 9, and 11 received ketogenic meals in addition to the ketogenic formulation (KetoCal® 2.5:1 LQ MCT Multi Fiber) during the whole or parts of the intervention, resulting in higher carbohydrate and protein intake. Days where patients were weaned off standard nutrition are not included in mean. E%: energy percent.

**Table 6:** Estimated daily nutrition intake at baseline in patients receiving full standard enteral nutrition before the intervention.

	Estimated daily nutrition intake at baseline and percent difference between baseline and 100% MCT-KD																	
Patient	Energy (kcal) Baseline	Energy (kcal) MCT-KD (% diff.)	Fat (g) Baseline	Fat (g) MCT-KD (% diff.)	Carbo-hydrate (g) Baseline	Carbo-hydrate (g) MCT-KD (% diff.)	Sugars (g) Baseline	Sugars (g) MCT-KD (% diff.)	Dietary Fiber (g) Baseline	Dietary Fiber (g) MCT-KD (% diff.)	Protein (g) Baseline	Protein (g) MCT-KD (% diff.)	Fat E% Baseline	Fat E% MCT-KD (% diff.)	Carbo-hydrate E% Baseline	Carbo-hydrate E% MCT-KD (% diff.)	Protein E% Baseline	Protein E% MCT-KD (% diff.)
**1**	1,950	12.5	73.6	184.8	234.0	-94.0	88.3	-88.4	3.9	256.4	86.0	-33.8	33.2	148.2	48.9	-92.4	17.8	-42.7
**2**	2,144	45.4	106.0	166.4	193.0	-84.9	34.4	-43.0	0.0	-	106.0	-4.5	43.5	86.7	36.5	-86.3	20.0	-34.5
**3**	1,666	38.4	83.3	162.3	146.0	-89.6	21.2	-47.6	0.0	-	83.3	-25.2	44.2	89.8	35.5	-88.7	20.3	-45.8
**4**	1,950	17.0	73.7	193.2	235.0	-93.5	99.0	-88.9	2.7	463.0	86.4	-28.1	33.2	152.4	48.9	-91.8	17.9	-38.0
**5**	-	-	-	-	-	-	-	-	-	-	-	-	-	-	-	-	-	-
**6**	-	-	-	-	-	-	-	-	-	-	-	-	-	-	-	-	-	-
**7**	1,950	1.1	73.6	155.2	234.0	-94.5	88.3	-89.5	3.9	228.5	86.0	-38.8	33.2	153.3	48.9	-92.0	17.8	-38.8
**8**	1,533	49.3	51.4	320.6	195.0	-92.0	19.5	-41.5	19.5	-21.0	61.2	3.3	29.7	181.5	54.1	-92.4	16.2	-30.9
**9**	1,672	46.9	71.5	219.6	154.0	-86.2	15.4	-3.9	0.0	-	103.0	-29.3	37.7	118.6	37.3	-87.4	25.0	-50.4
**10**	1,917	22.2	71.5	212.3	226.0	-93.4	29.8	-63.4	23.3	-35.6	82.5	-25.5	32.7	157.8	49.9	-92.2	17.4	-38.5
**11**	2,380	-4.3	119.0	76.4	208.0	-91.2	30.2	-60.3	0.0	-	119.0	-37.2	44.2	84.6	35.5	-87.6	20.3	-34.5
**12**	2,205	-10.4	83.2	125.0	263.0	-95.0	23.1	-58.9	30.3	-56.8	89.0	-39.8	33.1	152.9	50.6	-92.1	16.3	-31.9
**Mean**	**1,936**	**21.0**	**80.6**	**170.8**	**208.8**	**-91.3**	**44.9**	**-71.9**	**8.3**	**83.1**	**90.2**	**-22.5**	**36.4**	**126.4**	**44.6**	**-90.1**	**18.9**	**-36.5**

Patient 5 and 6 were orally fed and nutrition intake was not estimated. Mean percent difference between baseline and nutrition intake during the 100% ketogenic diet with added medium-chain triglycerides. E%: energy percent.

### Exploratory outcomes

The results of the functional measurements GCS, EFA, FIM, FOIS, and RLAS are seen in Table 7.

**Table 7:** Functional scores at admission to the Department of Brain Injury and after 4 weeks: Glasgow Coma Scale, Early Functional Abilities, Functional Independence Measure, Functional Oral Intake Scale, and Ranchos Los Amigos Scale for traumatic brain injury patients.

	Ketogenic diet group (*n* = 12)		Standard care reference group (*n* = 17)	
	Admission	4 weeks	Admission	4 weeks
Glasgow Coma Scale	12 (12 to 14)	14 (13 to 14) (*n* = 8)	13 (13 to 14)	14 (12 to 15) (*n* = 13)
Early Functional Abilities	44 (42 to 51) (*n* = 8)	73 (52 to 84) (*n* = 11)	59 (43 to 66) (*n* = 15)	79 (56 to 92) (*n* = 16)
Functional Independence Measure	20 (18 to 27) (*n* = 10)	32 (19 to 46) (*n* = 11)	23 (21 to 30) (*n* = 15)	45 (24 to 84) (*n* = 16)
Functional Oral Intake Scale	1 (1 to 3) (*n* = 11)	2 (1 to 4)	3 (1 to 4) (*n* = 14)	5 (2 to 7) (*n* = 13)
Ranchos Los Amigos Scale*	5 (4 to 6) (*n* = 3)	6 (5 to 7) (*n* = 3)	5 (4 to 5) (*n* = 2)	6 (6 to 7) (*n* = 2)

Data are presented as median (IQR).*Ranchos Los Amigos Scale was only measured in traumatic brain injury patients.

#### Bioelectrical impedance analysis and weight

The median weight change in the MCT-KD group from inclusion to completion/exclusion was 0.4 kg (interquartile range: -1.1 kg to 1.7 kg), and there were no significant body composition and weight changes in the patients during the first 2 (*n* = 12) and 5 (*n* = 9) weeks (Supplementary Tables 4 and 5).

## Discussion

### Feasibility outcomes

#### Recruitment of patients

Being the first study in subacute ABI, the results can only be compared with the five previous studies examining a KD in acute ABI (9, 16–19). For the recruitment feasibility criteria to be fulfilled, we set a goal of ≥60% eligible patients to be included, based on a trial in acute TBI patients (25, 37) using a lower limit of the 95% confidence interval of ≥60% included eligible patients. This trial by Riberholt et al. (25, 37) included 76% [95% CI: 63% to 86%] of eligible patients. In our study, 13 out of 30 patients were eligible (43% [95% CI: 27% to 61%]), and 12 of the eligible patients were included (92% [95% CI: 67% to 99%]). We, therefore, surpassed our goal of including ≥60% eligible patients by 32%.

In the study by Arora et al. (18) 10 out of 72 patients were found eligible (14% [95% CI: 8% to 24%]), and all eligible patients were included (100% [95% CI: 72% to 100%]). Bernini et al. (9) screened 42 patients, of which 20 were eligible (48% [95% CI: 33% to 62%]), and all eligible patients were included in the study (100% [95% CI: 72% to 100%]). White et al. (19) and Robertson et al. (17) do not specify how many patients were screened and found eligible, and Bernini et al. (9) included patients from two studies with no further details about screening. From the results of this study and the studies by Arora et al. (18) and Bernini et al. (9) 14% to 48% of screened patients were eligible, and 92% to 100% of eligible patients were included. Once the eligible patients were found, it was the impression of the investigator that next of kin was eager to consent on behalf of the patient and several patients had participated in several trials since admission to the ICU.

As the primary outcome of this study was feasibility, we chose to include ABI patients with different diagnoses. White et al. (19) included both patients with TBI (*n* =15), SAH (*n* =2), and stroke (*n* = 3), whilst Bernini et al. (9) and Arora et al. (18) included TBI patients only. The age group in our study and the studies by Bernini et al. (9), Arora et al. (18) and White et al. (19) were similar. White et al. (19) only included patients with severe ABI (GCS at admission 3 to 4), whilst our study and the studies by Bernini et al. (9) and Arora et al. (18) included patients with severe-to-moderate ABI (GCS at admission from 3 to 14).

We included a SCR group as a reference primarily for safety reasons to investigate if the occurrences of SAEs and AEs not considered serious, were higher in the MCT-KD group. We conclude that the incidence of SAEs and AEs not considered serious were almost identical in the two groups. Another reason we included a reference group was the possibility of performing a power calculation for a future trial investigating the effect of MCT-KD in subacute ABI. However, the patients included in the SCR group were predominantly patients not eligible for the study. At admission, GCS was similar in both groups, but baseline EFA, FIM, and FOIS were better in the SCR group. The SCR group comprised patients in both a potentially better and worse state than those in the MCT-KD group. Due to the small sample size and missing data, statistical analysis was not performed on the exploratory outcomes EFA, FIM, FOIS, and RLAS.

#### Attaining and maintaining ketosis

For the attaining ketosis criteria to be fulfilled, we set a goal of ≥75% of patients attaining ketosis within 5 days from initiating 100% MCT-KD, based on the acute ABI studies by Arora et al. (18) and White et al. (19). For the maintaining ketosis criteria, we set a goal of ≥75% of days in ketosis since ketosis was first obtained, based on a study by McDonald et al. (26) in epilepsy patients. In our study, it took a median of 1 day to achieve ketosis from starting 100% MCT-KD, and it was maintained for 97% of the time after ketosis was attained, meaning that both goals were achieved.

The five previous studies in acute ABI have either used enteral nutrition (9, 16, 18, 19) or parenteral nutrition (17). The enteral nutrition products used in previous studies and in our study are different, and the macronutrient compositions differ, which makes the results difficult to compare. Including patients receiving ketogenic meals, the mean energy intake in this study was 82.4% fat, 4.4% carbohydrate, and 12.0% protein, which is the closest to the macronutrient composition in the study by White et al. (19). For future studies of efficacy, it is important to standardise the nutrition protocol to be able to compare the results between studies.

An increase in b-BHB and a decrease in BG was observed in our study over 6 weeks (Figure 2), similar to the observations over a 6-day period by White et al. (19). In our study, mean b-BHB increased ≥0.5 mmol/L within 1 day, and BG decreased over 2 weeks before stabilising for 4 weeks. In the study by Arora et al. (18) serum BHB levels >0.27 mmol/L were achieved within a mean of 2 days and were maintained in eight out of 10 patients between 4 and 14 days. In the study by Ritter et al. (16) b-BHB increased in the intervention group whilst BG remained unchanged at fasting level. This could be due to the nutrition given to the intervention group containing 0 g of carbohydrate (16). The b-BHB and AcAc also increased in the study by Robertson et al. (17) where parenteral nutrition was given, and BG decreased from day 1 and stabilised from day 3 to 5.

In the study by Bernini et al. (9) levels of plasma BHB and AcAc decreased from fasting to stable nutrition state, which was likely due to the higher carbohydrate content (13.5 g carbohydrate/100 mL) in the nutrition they used. The study by Bernini et al. (9) found a modest but statistically significant increase in plasma C8 and C10 with a high-level MCT enteral formula (MCT-enriched Peptamen AF®, Nestlé), but this level of medium-chain fatty acid did not result in an increase of plasma KB level or a greater brain KB level at stable nutritional state. This might be due to the MCT formulation containing 13.5 g carbohydrate/100 mL, which is not ketogenic (MCT 23 g/1,000 kcal). The authors conclude that the administration of MCT by continuous enteral feeding is ineffective in raising KB to therapeutically relevant levels. This study shows that a ketogenic MCT formulation (KetoCal® 2.5:1) containing 1.1 g carbohydrate/100 mL can raise b-BHB to therapeutically relevant levels (approximately MCT 25.4 g/1,000 kcal). The levels of C8, C10, and BHB in the brain were not measured in this study.

In previous acute ABI studies, it is not stated at what time of day BHB and BG levels were measured and if the patients were in a fasted or postprandial state. This should be considered when comparing the results of these studies.

An important effect of ketogenic interventions in ABI, besides providing an alternate energy substrate for the brain, is glycaemic control. Especially in the acute phase of ABI, maintaining control of BG is crucial. Hyperglycaemia compromises microcirculatory blood flow, increases blood–brain barrier permeability, promotes inflammation, and triggers osmotic diuresis, hypovolemia, and immunosuppression, which harms the injured brain further. Intensive insulin therapy is the standard of care to control BG but is associated with unacceptable rates of hypoglycaemia and metabolic crisis (38). In a study by Svart et al. (39) infusions of 3-hydroxybutyrate in healthy subjects showed that cerebral glucose utilisation decreased by 14%, oxygen consumption remained unchanged, and cerebral blood flow increased by 30% in all measured brain regions. The authors propose that increased oxygen supply concomitant with unchanged oxygen utilisation may contribute to the neuroprotective effects of KB. There is consensus that hyperglycaemia in the acute phase of ABI is detrimental to the injured brain, and the current standard of care in acute ABI is to give patients i.v. fluids free from glucose, but at the same time, the enteral nutrition formulations given to patients as standard of care, contains high amounts of carbohydrate and sugars, which is contradictory.

In this study, we used a ketogenic enteral formulation and also included ketogenic meals and a large variety of ketogenic and low-carb food products in the intervention, and still succeeded in attaining and maintaining ketosis. The extensive occupational therapy at this stage of neurorehabilitation requires a wide variety of food items and consistencies both for the assessment of dysphagia and swallowing abilities and for training patients to regain the ability to eat, drink, cook, and prepare food again. This study succeeded in maintaining the MCT-KD despite these challenges. The intervention period in this study was 6 weeks, whilst the intervention in previous studies in acute ABI (9, 16–19) varied between 3.5 days and 2 weeks, and still ketosis was maintained in this study.

Another strength is that b-BHB and BG were measured three times daily with FreeStyle Precision Neo® (Abbott Laboratories A/S, Copenhagen, Denmark), and only 5% of measurements were missed. Furthermore, FreeStyle Precision Neo® meets the accuracy criteria in ISO 15197: 2013 standard (40, 41) and has been tested for accuracy in other trials (42–44). ISO 15197: 2013 does not specify the accuracy of b-BHB measurements. It should be noted that the accuracy of BG measurements may be affected by biochemical changes that often occur in critically ill patients and by the medication they receive. Little is known about the influence of these factors on b-BHB measurements (45).

Our study and previous studies show that it is possible to induce ketosis in both acute and subacute ABI patients within days (16–19). Compliance and maintenance of ketosis will be easier in patients receiving full enteral or parenteral feeding, but we demonstrate that even with patients capable of oral nutrition and conscious enough to decline the meals, it is possible to attain ketosis fast and maintain it for 6 weeks. It is hypothesised that achieving ketosis quickly in acute ABI is important in limiting the secondary injury cascade, but as this research is still in its early phases, this still needs to be examined. In subacute ABI, the secondary injury cascade has already occurred for some time, and it is probable that if ketosis has an effect during this phase of ABI, the rate of attaining ketosis is of less importance, although maintaining ketosis during a period of time might be more important.

#### Occurrence of serious adverse reactions and adverse reactions to MCT ketogenic diet

For the occurrence of SARs and ARs to the MCT-KD, we set a goal of ≤5% of intervention days with SARs in 100% of patients and ≤ 30% of intervention days with ARs in ≥75% of patients based on the results by Arora et al. (18) and White et al. (19). In this study, there were no SARs to the MCT-KD and 9.5% of intervention days with ARs in total, and therefore the occurrence of SARs and ARs to the MCT-KD were within the predefined goals.

In previous studies in acute ABI reporting on ARs, White et al. (19) described eight episodes of vomiting and diarrhoea and no episodes of hypoglycaemia or seizures. One patient developed metabolic acidosis during severe sepsis, malnutrition, and vasopressor requirements. The anion gap and b-BHB returned to normal within 36 h after the ketogenic feed was stopped.

In the study by Arora et al. (18) mild abdominal pain was reported in one patient, hypertriglyceridaemia in two patients, and hypoglycaemia in one patient. In Cochrane reviews from 2018 and 2020 by Martin-McGill et al. (46, 47) examining the KD for drug-resistant epilepsy, the most commonly reported ARs to the KD were gastrointestinal symptoms (vomiting, constipation, and diarrhoea). The gastrointestinal ARs were more frequent with the classic 4:1 ratio KD than the more moderate KD. This is consistent with our results, where 8.3% out of 9.5% of intervention days with ARs were due to nausea (3%), vomiting (2%), and diarrhoea (3.3%).

One patient treated repeatedly with antibiotics for urinary tract infection before and during the intervention had 11 days with diarrhoea during the intervention. One patient was diagnosed with *C. difficile* twice during the intervention and had the infection for 2 days with diarrhoea and one episode with stomachache. One patient experienced nausea whilst diagnosed with severe hydrocephalus. When occurring during the intervention, the above are interpreted as ARs not considered serious to the MCT-KD. A patient with Horton’s disease and frequent severe migraines had one episode of headache on day 1 and no headache episodes for the rest of the 6-week intervention. Ten out of 13 episodes (77%) with nausea and six out of nine (67%) episodes with vomiting were in patients having a nasogastric tube at that time. It is possible that this was due to the faster administration of enteral nutrition to patients with a nasogastric tube, for whom it was given as a bolus (<15 min), compared to patients with a percutaneous endoscopic gastrostomy, where enteral nutrition was continuously infused over a few hours per meal (2–3 h).

There are many confounders affecting the results of this study. The patient group is very complex and heterogeneous, and many patients had several comorbidities (Supplementary Table 2), which can give similar symptoms to all registered ARs in this study. A wide variety of medications commonly prescribed to this patient group have many adverse effects, which can have affected the outcome. Patients also recieved medication to stop diarrhoea and antiemetic medications to treat nausea. Commonly occurring drops in blood pressure can cause feelings of dizziness, nausea, and, in some cases, vomiting. Due to the many factors mentioned above, it is not possible to know whether the ARs reported in this study were caused by the MCT-KD, if they were AEs caused by other factors, or whether some of the reported AEs were actually ARs. Moreover, it is possible that ARs from the MCT-KD were stopped or obscured by painkillers and medication that treated diarrhoea and nausea, and, therefore, was never registered.

In the study by Ritter et al. (16) plasma triglycerides increased in the patients on the experimental diet and were significantly higher than in the control diet group on days 5 to 14. On day 14, plasma triglycerides averaged 3.32 ± 0.62 mmol/L (converted to SI units for comparison) in the experimental diet group, compared to 1.31 ± 0.18 mmol/L in the patients on the control diet. The highest concentration of plasma triglycerides that was observed with the experimental diet was 5.15 mmol/L. The plasma triglycerides reported by Ritter et al. (16) are consistent with the plasma triglycerides results in this study, with the exception that the highest observed plasma triglycerides in this study were >10.0 mmol/L in the diabetes mellitus type 2 patient. This patient’s plasma triglycerides were 4.10 mmol/L at inclusion. Approximately 60% of diabetes mellitus type 2 patients are also diagnosed with non-alcoholic fatty liver disease, where the production of KB progressively reduces, whilst hepatic glucose synthesis and output increase (48). Non-diagnosed non-alcoholic fatty liver disease in this patient could potentially explain the elevated plasma triglycerides. In this study, plasma triglycerides were measured postprandial in all patients after receiving a high fat meal. The high levels of triglycerides in circulation after high fat meals could partly explain the elevated levels of plasma triglycerides in some patients. It would have been optimal to measure fasting levels of plasma triglycerides in all patients to eliminate the effect of the previous meal, but that would mean a delay in feeding. In the diabetes mellitus type 2 patient, fasting plasma triglycerides were measured after day 24 to get a more accurate result.

The KD is known to increase urinary excretion of electrolytes, especially sodium and potassium (49, 50). Hyponatraemia is common in patients with ABI for several reasons (35, 36, 51), but little is known about how ketosis affects plasma sodium status in ABI patients. The lack of knowledge about the KD’s effects in this patient group meant that there was no protocol on sodium chloride supplementation. The prescription of sodium chloride supplements to patients was inconsistent and dependent on the patient’s physician. Even with dosages up to 10 g of sodium chloride a day, there were episodes of hyponatraemia in some patients, whilst other patients had normal plasma sodium without sodium chloride supplements.

A strength of this study is the safety monitoring during the intervention of 6 weeks where potential adverse effects could occur, and still they were not more prevalent than in previous acute ABI studies (9, 16–19) with a duration between 3.5 days and 2 weeks. There is still little safety data on ketogenic interventions in ABI patients. The SARs and ARs in previous acute ABI studies (9, 16–19) and in this subacute ABI study are congruent with the epilepsy literature (46, 47). More research is needed to be able to conclude if ketogenic interventions are safe in acute and subacute phases of ABI.

#### Acceptance of treatment and transitioning from enteral feeding to oral intake

For the acceptance of treatment criteria, we set a goal of ≥75% of patients accepting the treatment in ≥75% of intervention days (26). In previous studies, patients were either in a coma or had very low consciousness (GCS on admission: 3 (19), 5.6 (16), 6 (9), < 8 (17, 18)), which makes compliance easier, as most patients received enteral feeding only. In this study, ketogenic meals and a wide variety of low-carb food products were used as snacks and in occupational training. Two patients were exclusively orally fed, and one patient received both enteral feeding and ketogenic meals. All patients accepted the MCT-KD in 100% of intervention days. For the transitioning from enteral feeding to oral intake criteria, we set the goal of ≤30% of intervention days with complications in ≥75% of patients transitioning from enteral to oral intake (27). There were no complications related to transitioning from enteral feeding to oral intake.

## Conclusion

To the best of our knowledge, this feasibility study is the first clinical human trial examining a KD in patients with subacute ABI. All six predefined feasibility criteria were achieved, and we therefore conclude that a MCT-KD intervention is feasible for 6 weeks in hospitalised adult patients with subacute ABI. Recruitment of patients was efficient, ketosis was achieved rapidly and maintained during the intervention, there were no SARs, the occurrence of ARs were low, the MCT-KD was accepted by all participants, and there were no complications when transitioning from enteral to oral feeding.

### The implications for future research

The knowledge obtained in this study is important for planning future studies with the KD in this patient group. Knowing that this intervention is feasible with respect to the chosen parameters, future RCTs can be planned to examine the effects of a KD on adult patients with subacute ABI. Due to the complexity of the patient group with many comorbidities and confounders affecting the results, future RCTs must be carefully planned, and the sample size must be powered to examine a possible effect of the KD on ABI. To better be able to compare the results of future studies, it is important to standardise ketogenic nutrition protocols both in animal and human studies. Another important aspect is to investigate the feasibility and efficacy of different formulated KDs with and without MCT supplementation and exogenous ketones, and also investigate these supplementations with other types of diets. Similar to ketone therapy, lactate therapy with exogenous supplemental lactate can be effectively utilised by the injured brain to boost cerebral energy metabolism and potentially reduce the secondary injury cascade of neurodegenerative processes (7, 38, 52). For future studies, ketone and lactate therapy should be investigated separately and in combination.

## Data availability statement

The original contributions presented in the study are included in the article/Supplementary material, further inquiries can be directed to the corresponding author.

## Ethics statement

The study involving humans were approved by Capital Region’s Committee on Health Research Ethics, Copenhagen, Denmark (H-20018775). The study was conducted in accordance with the local legislation and institutional requirements. Written informed consent for participation in this study was provided by the participants or the participants’ legal guardians/next of kin in the case of patients without capacity to consent.

## Author contributions

ME: Conceptualization, Data curation, Formal analysis, Funding acquisition, Investigation, Methodology, Project administration, Resources, Validation, Visualization, Writing – original draft, Writing – review & editing. JA: Conceptualization, Methodology, Supervision, Writing – review & editing. DC: Data curation, Formal analysis, Visualization, Writing – review & editing. CR: Data curation, Formal Analysis, Methodology, Writing – review & editing. IP: Conceptualization, Methodology, Project administration, Supervision, Writing – review & editing.
