# Supplementary material for: Diet-induced ketosis in adult patients with subacute acquired brain injury: a feasibility study
Source: Front Med (Lausanne). 2024 Mar 19;10:1305888. doi: 10.3389/fmed.2023.1305888 (PMC10990248; doi:10.3389/fmed.2023.1305888)
Supplement: Supplementary file 1 [file Data_Sheet_1.pdf]

Supplementary Material

Table of contents

Supplementary Table 1. Contraindication to a Ketogenic Diet 3
Supplementary Table 2. Patients' comorbidities and previous diagnosis................................. 4
Supplementary Table 3. Specification of serious adverse events and adverse events 6
Supplementary Figure 1. Number of days in ketosis and out of ketosis in patient 1-12 7
Supplementary Figure 2 – 13. Blood β-hydroxybutyrate and blood glucose in patient 1-12 8
Supplementary Figure 14. Plasma Triglycerides in patient 1-12 14
Supplementary Table 4 and 5. Changes in Body Weight and Body Composition 15
Supplementary Table 6. CONSORT 2010 checklist of information to include when reporting a pilot or feasibility trial 17
Supplementary Table 7. TIDieR (Template for Intervention Description and Replication) Checklist 19

Supplementary Table 1. Contraindication to a Ketogenic Diet

Contraindication to a Ketogenic Diet
Absolute contraindications
Carnitine deficiency (primary)
Carnitine palmitoyl transferase (CPT) I or II deficiency
Carnitine translocase deficiency
β-oxidation defects
Medium-chain acyl dehydrogenase deficiency (MCAD)
Long-chain acyl dehydrogenase deficiency (LCAD)
Short-chain acyl dehydrogenase deficiency (SCAD)
Long-chain 3-hydroxyacyl-CoA deficiency
Medium-chain 3-hydroxyacyl-CoA deficiency
Pyruvate carboxylase deficiency
Porphyria
Relative contraindications
Inability to maintain adequate nutrition
Propofol current use (risk of propofol infusions syndrome may be higher)

Table is modified from Kossoff EH, Zupec-Kania BA, Auvin S, Ballaban-Gil KR, Christina Bergqvist AG, Blackford R, et al. Optimal clinical management of children receiving dietary therapies for epilepsy: updated recommendations of the international ketogenic diet study group. *Epilepsia Open*. (2018) 3:175–92. doi: 10.1002/epi4.12225

Supplementary Table 2. Patients' comorbidities and previous diagnosis

**Comorbidities and previous diagnosis**	**MCT - Ketogenic Diet group** (number of patients)	**Standard Care Reference group** (number of patients)
Hydrocephalus diagnosed at the intensive care unit	2	1
Previous cerebral aneurysm	1	-
Previous multiple traumatic brain injuries	1	-
Hypertension	3	6
Hypercholesterolaemia	1	1
Diabetes Mellitus type 2	-	1
Class III obesity	-	1
Ischemic heart disease	1	2
Pacemaker due to diseased sinus node	1	1
Pacemaker due to other cause	-	1
Mechanical aortic valve replacement	1	1
Internal carotid artery stenosis	-	1
Atrial fibrillation	1	-
Third-degree atrioventricular block	-	1
Angina Pectoris	-	1
Cardiac arrest after current traumatic brain injury	1	-
Hypothyroidism	1	-
Osteoporosis	1	-
Cholesteatoma	1	-
Horton's disease	1	-
Scheuermann's disease	1	-
Severe migraine	1	-
Bechterew's disease	1	-
Epilepsy	1	-
Depression	1	1
Generalized anxiety disorder	-	1
Alcohol use disorder	2	4
Borderline personality disorder	1	-
Post-Traumatic Stress Disorder	-	1
Dissociative disorder	-	1
Hallucinations	-	1
Cholesystectomy	1	-
Subconjunctival eczema and glaucoma	1	-

Chronic obstructive pulmonary disease	1	-
Arthritis urica	1	-
Rheumatoid arthritis	-	1
Chronic backpain	1	1
Sleep apnea	1	-
Asthma	1	-
Previous Hodgkin's lymphoma	1	-
Previous prostate cancer	1	-
Previous disc herniation	1	-
Previous breast cancer	1	-
Previous oropharyngeal cancer	-	1
Ongoing evaluation for Parkinson’s disease	1	-
Deaf one ear	1	-
Blind one eye	-	1
Hearing impairment	-	1
Sarcoidosis	-	1
Uveitis	-	1
Hiatus hernia	-	1
Gastroesophageal reflux disease	-	1
Gingivitis	-	1
Psoriasis	-	2

Supplementary Table 3. Specification of serious adverse events and adverse events

	Ketogenic Diet group (n = 12)	Standard care reference group (n = 17)	Comments
**Serious adverse events**			
Epilepsy seizure	0	1	
Psychosis	0	2	
Hydrocephalus (Ventriculo-peritoneal/atrial shunt)	1	1	
Pneumonia	3	4	
Deep venous thrombosis / Pulmonary embolism	1	1	
Urinary tract infection	8	11	
**Total**	**13**	**20**	

Adverse events not considered serious	Ketogenic Diet group (n = 12)	Standard care reference group (n = 17)	Comments
Pressure wound ≥ 1	2	0	
Agitation	0	1	
Hearing loss	1*	0	*Due to earwax
**Total**	**3**	**1**	

Supplementary Figure 1. Number of days in ketosis and out of ketosis in patient 1-12

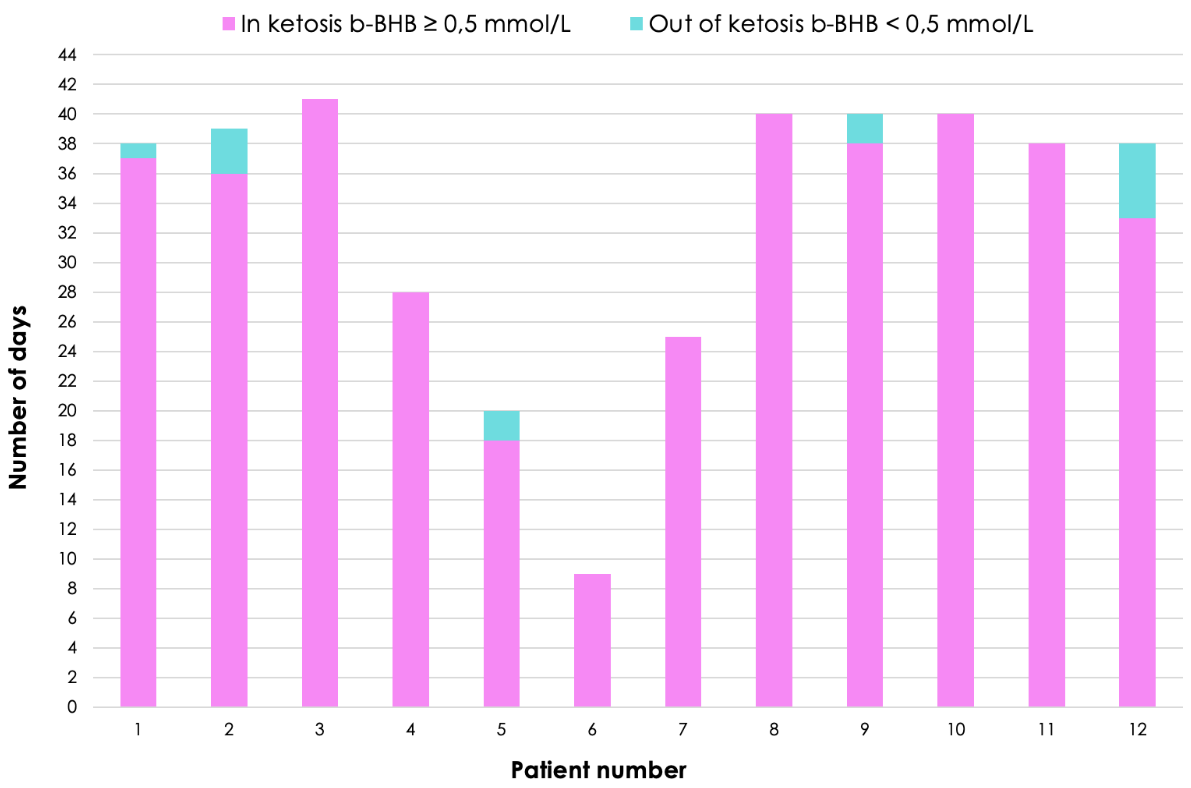

Figure 1 Number of days for each patient in ketosis or out of ketosis. A day in ketosis was defined as a mean value of the three-daily β-hydroxybutyrate measurements ≥ 0,5 mmol/L.

Supplementary Figure 2 – 13. Blood β-hydroxybutyrate and blood glucose in patient 1-12

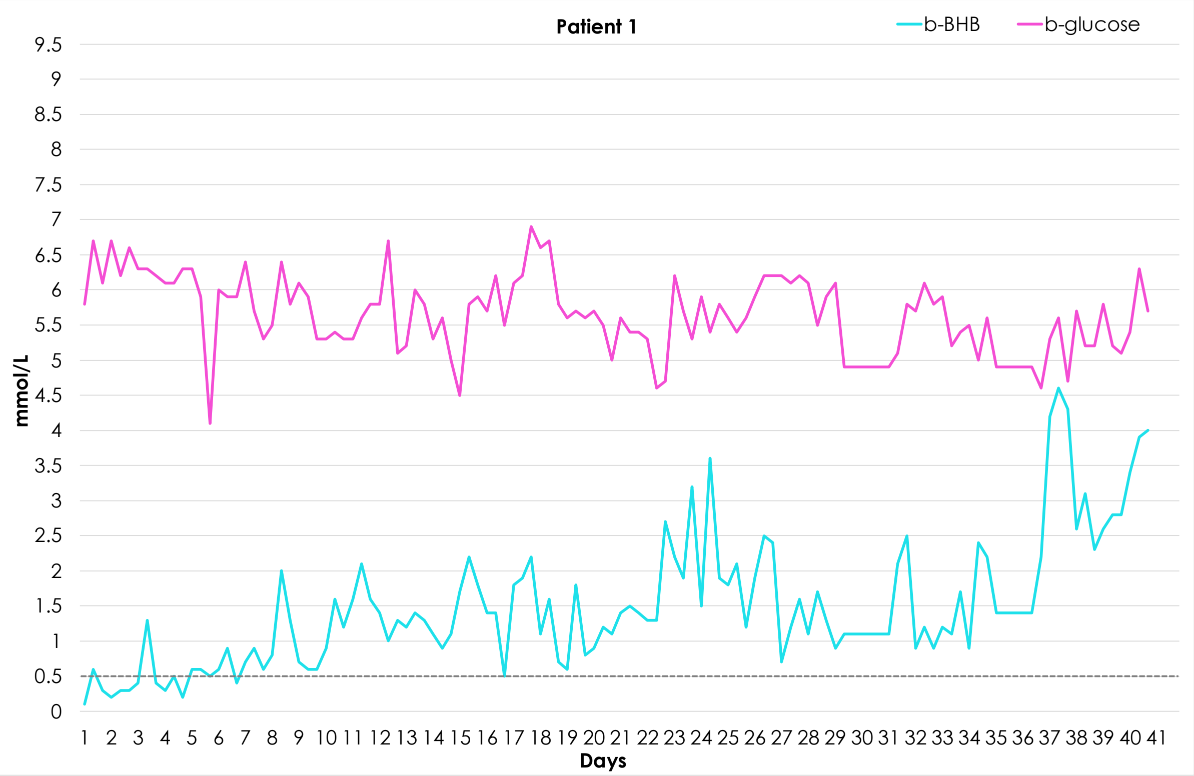

Figure 2 β-hydroxybutyrate and blood glucose levels in **patient 1** during intervention.

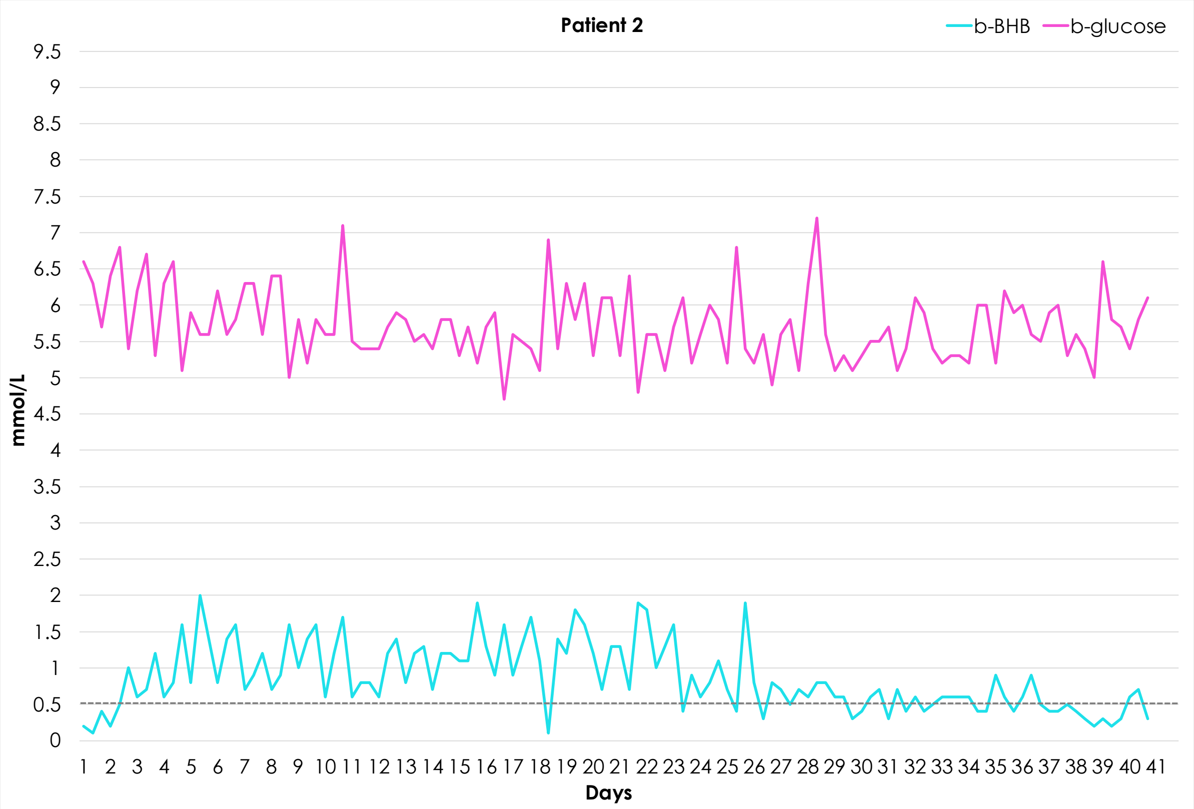

Figure 3 β-hydroxybutyrate and blood glucose levels in **patient 2** during intervention.

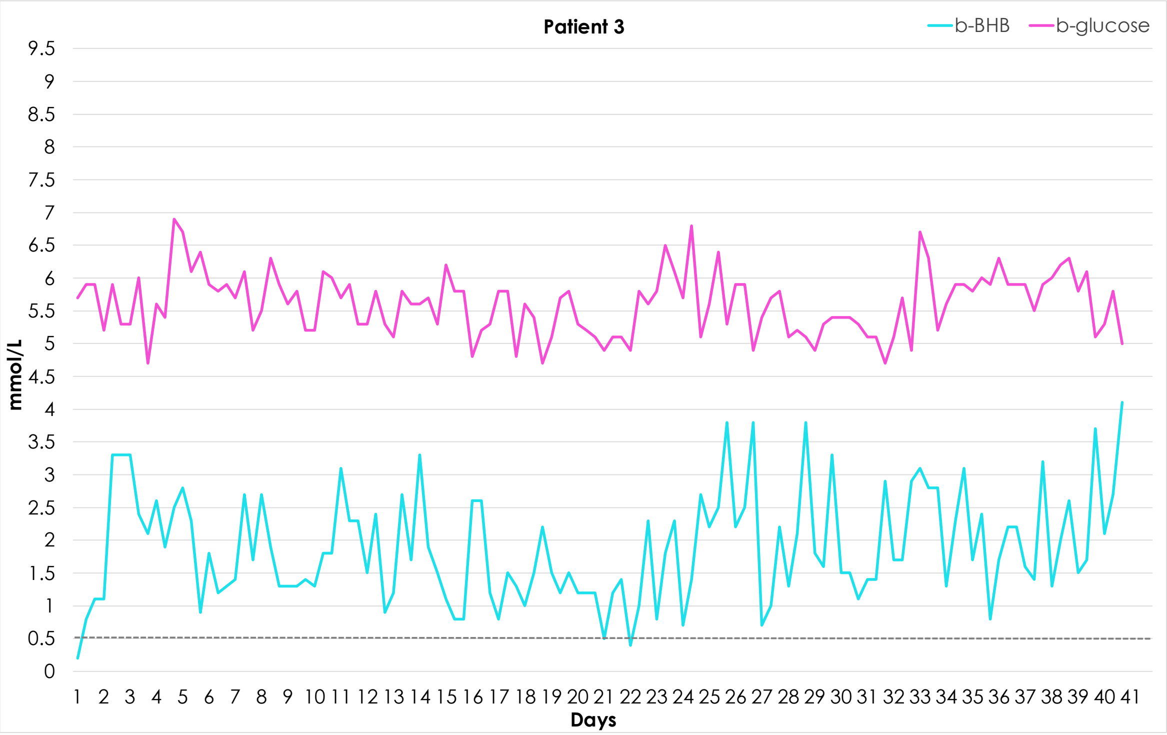

Figure 4 β-hydroxybutyrate and blood glucose levels in **patient 3** during intervention.

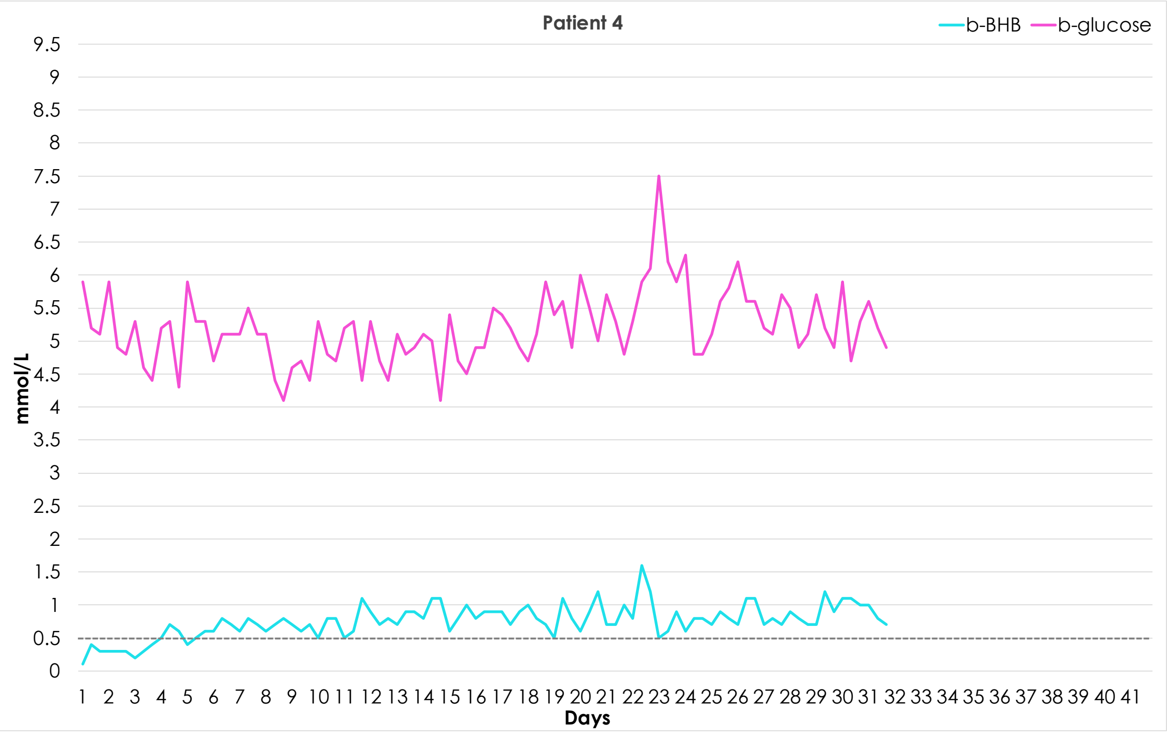

Figure 5 β-hydroxybutyrate and blood glucose levels in **patient 4** during intervention.

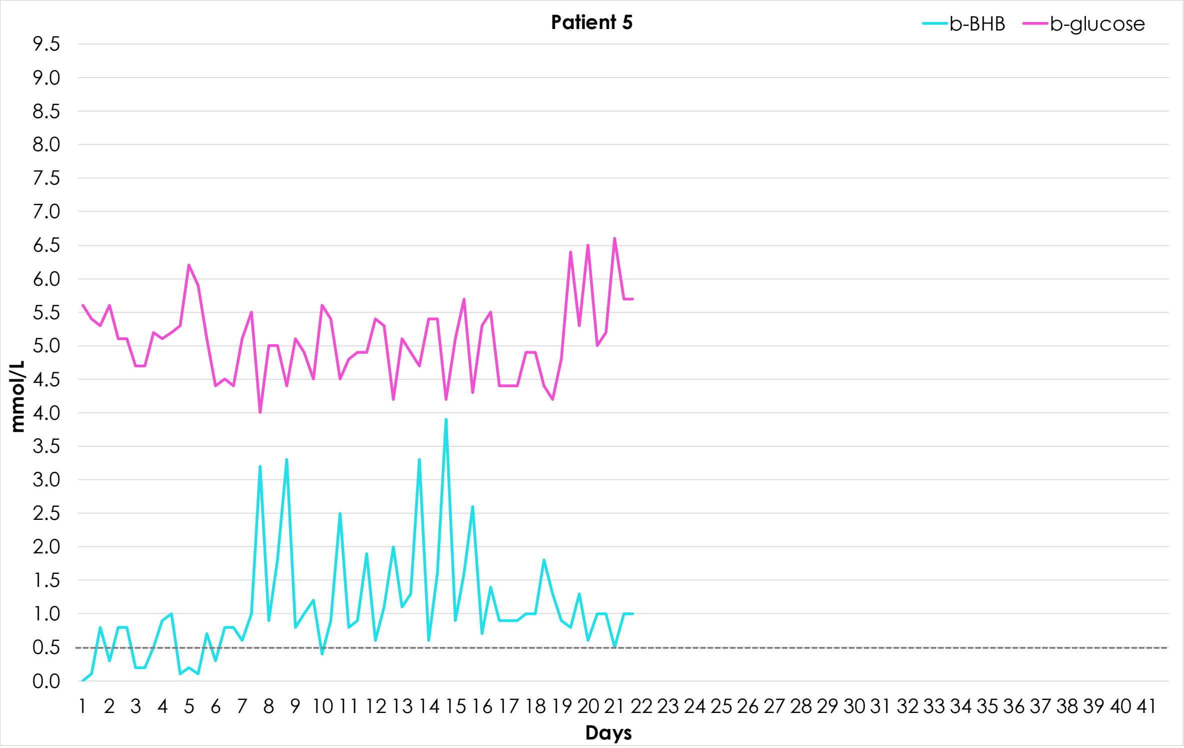

Figure 6 β-hydroxybutyrate and blood glucose levels in **patient 5** during intervention.

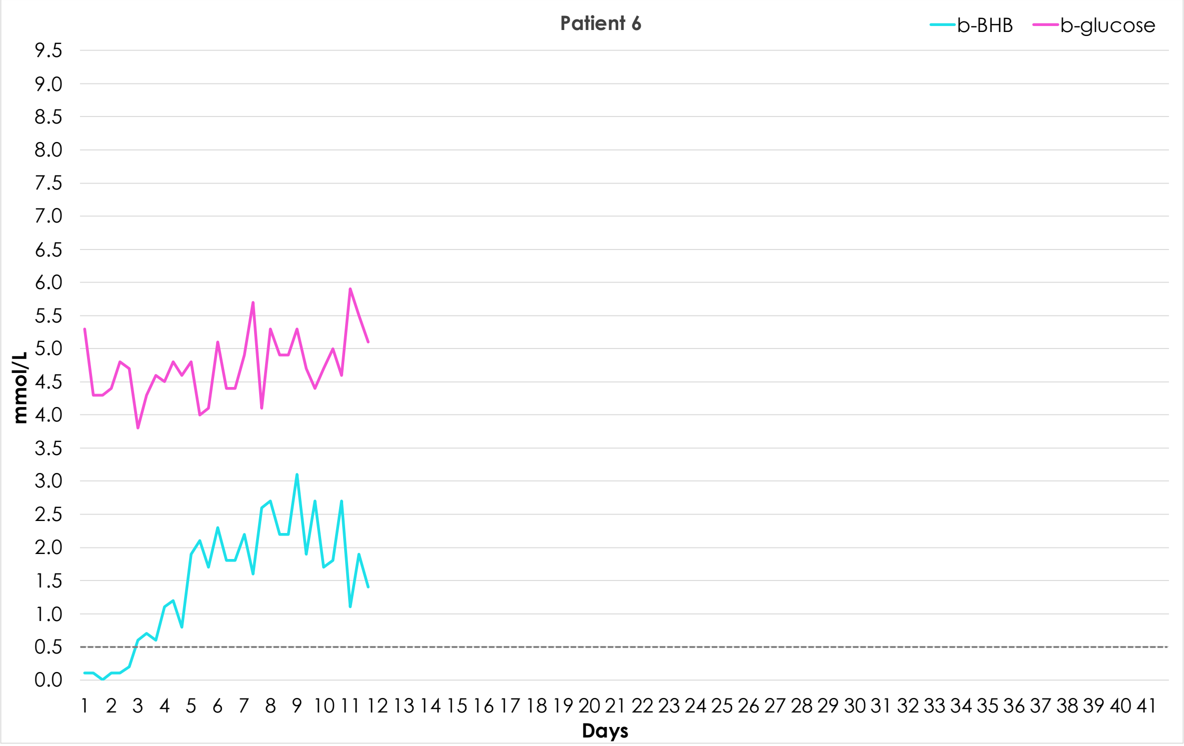

Figure 7 β-hydroxybutyrate and blood glucose levels in **patient 6** during intervention.

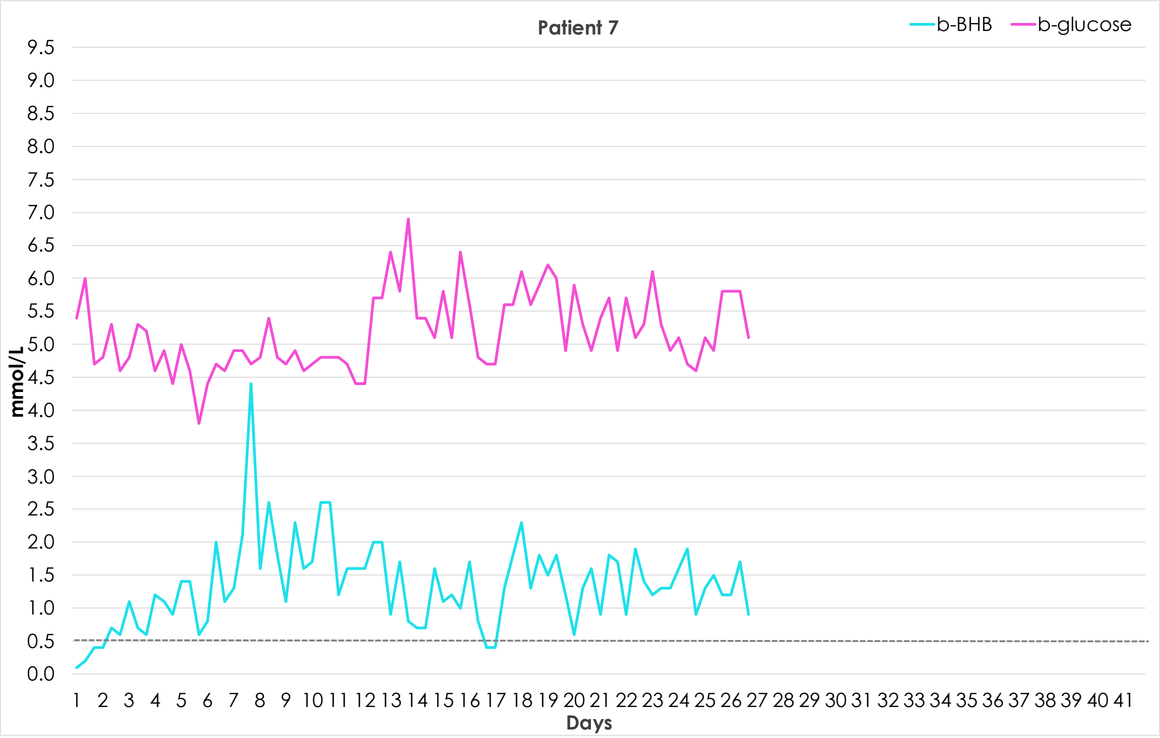

Figure 8 β-hydroxybutyrate and blood glucose levels in **patient 7** during intervention.

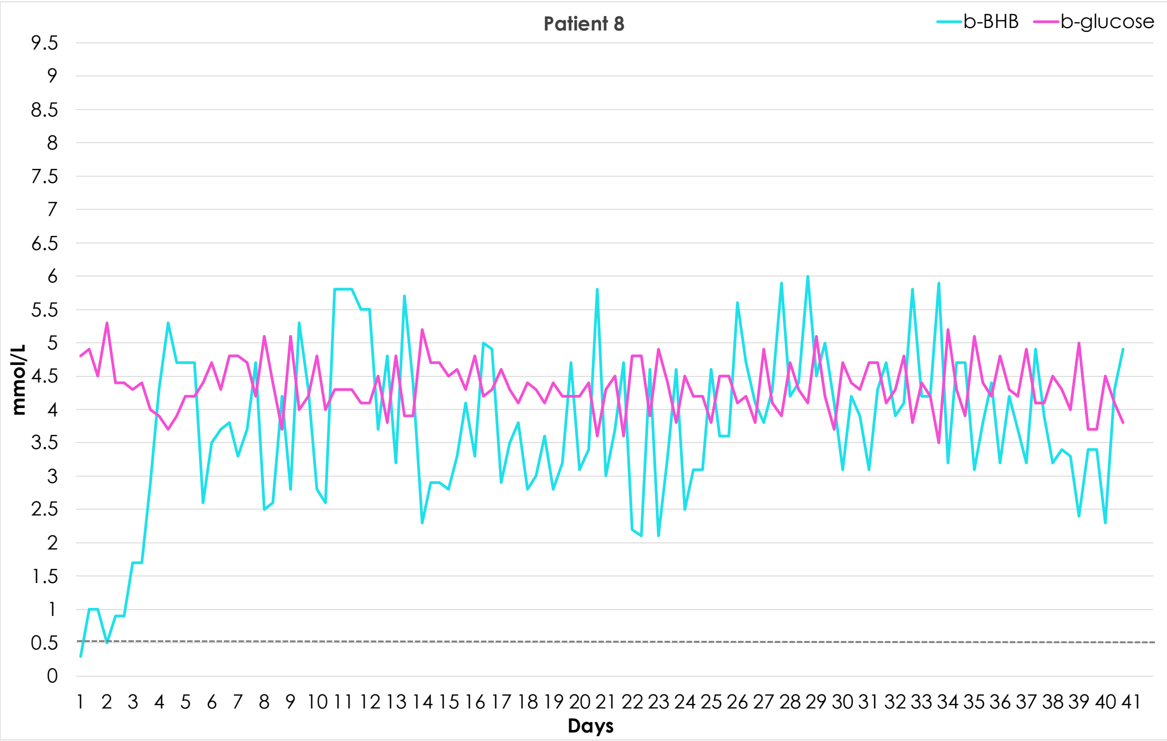

Figure 9 β-hydroxybutyrate and blood glucose levels in **patient 8** during intervention.

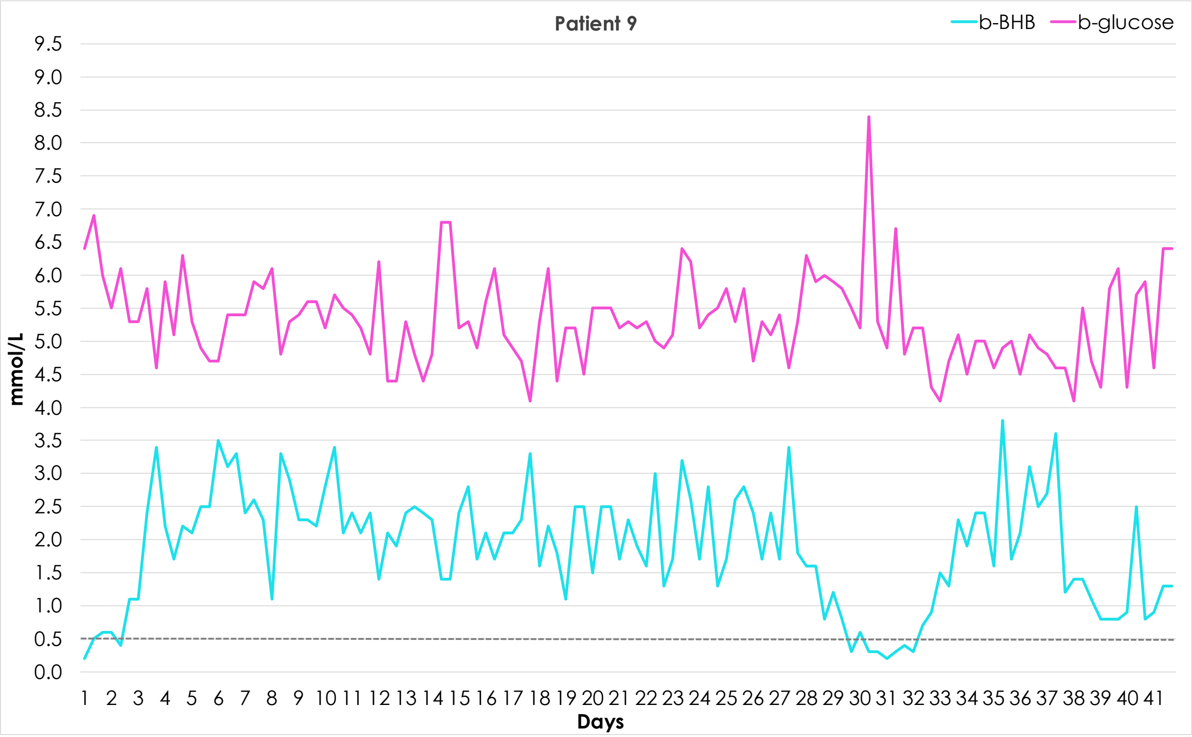

Figure 10 β-hydroxybutyrate and blood glucose levels in **patient 9** during intervention.

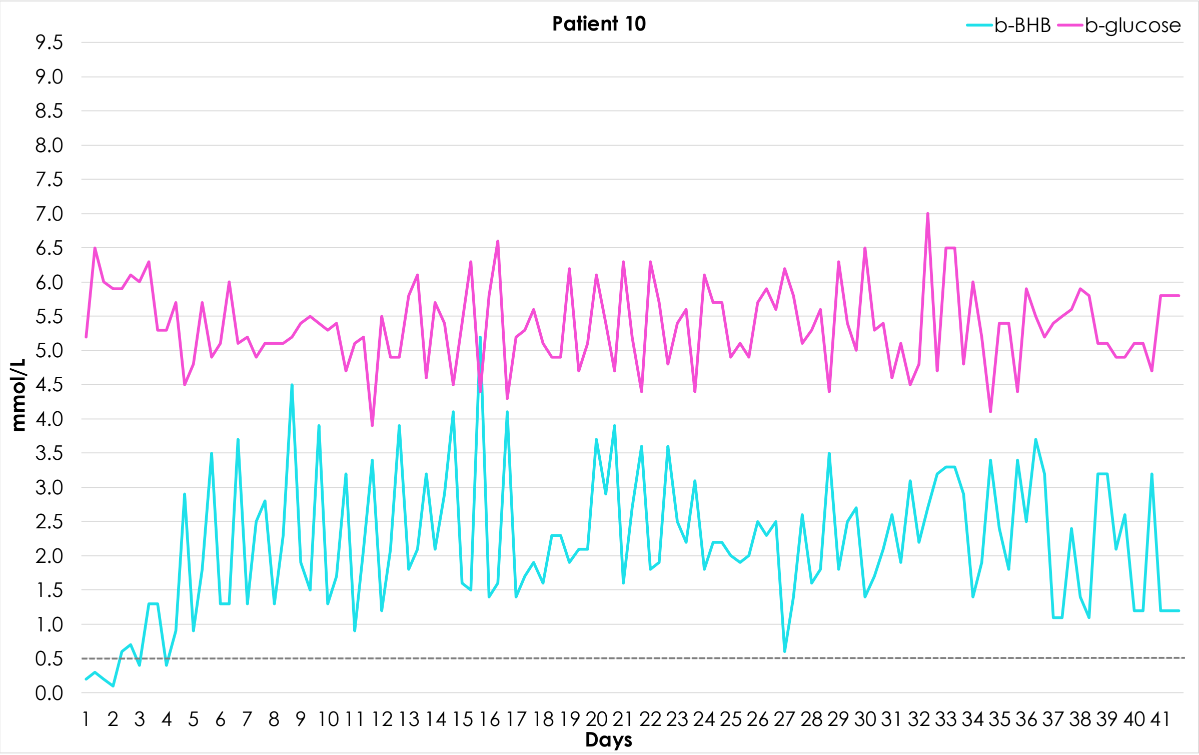

Figure 11 β-hydroxybutyrate and blood glucose levels in **patient 10** during intervention.

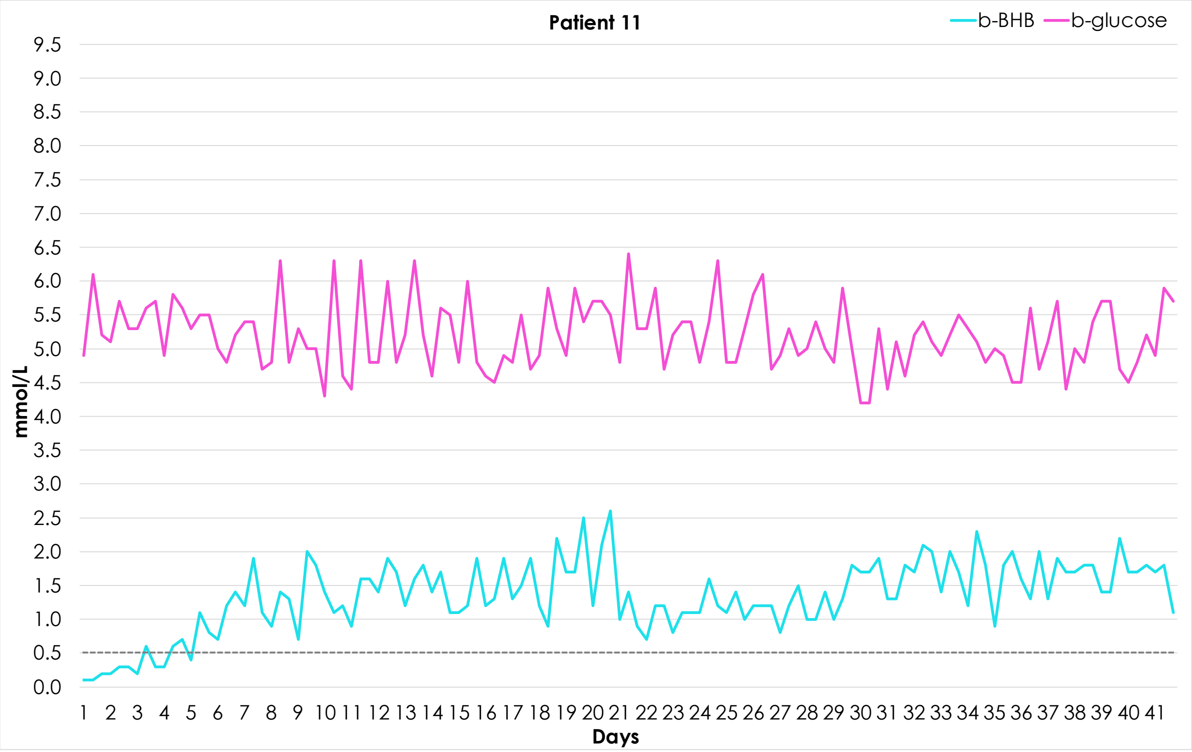

Figure 12 β-hydroxybutyrate and blood glucose levels in **patient 11** during intervention.

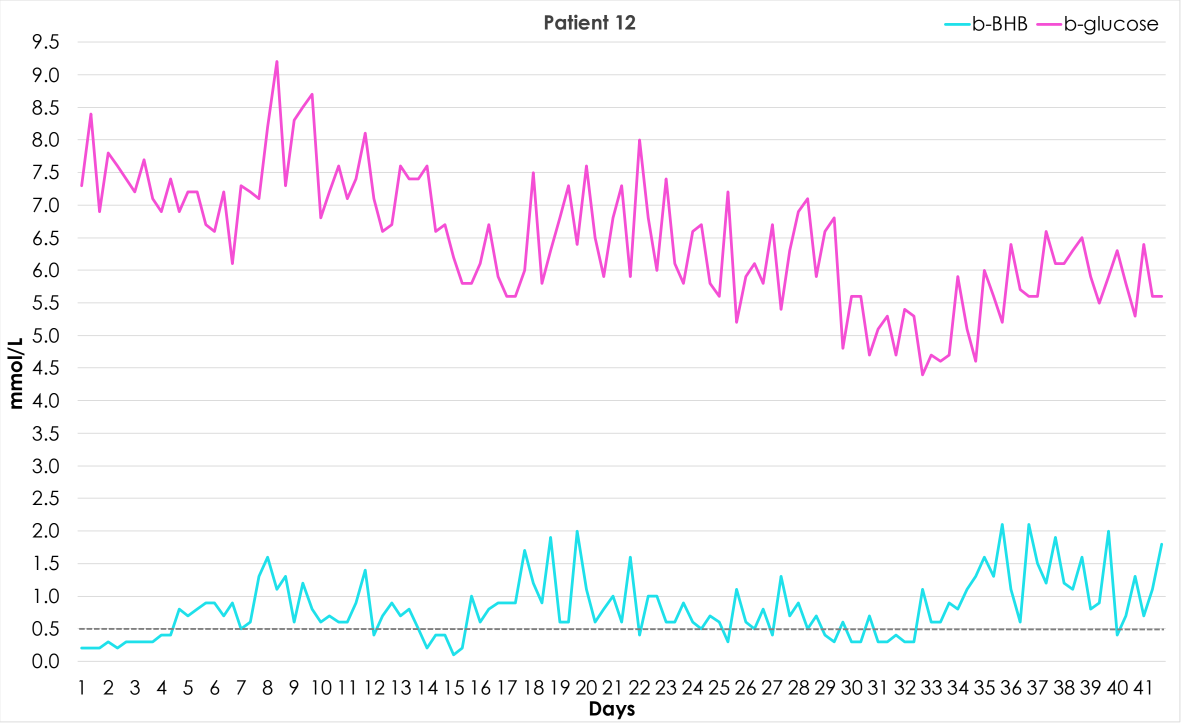

Figure 13 β-hydroxybutyrate and blood glucose levels in **patient 12** during intervention.

Supplementary Figure 14. Plasma Triglycerides in patient 1-12

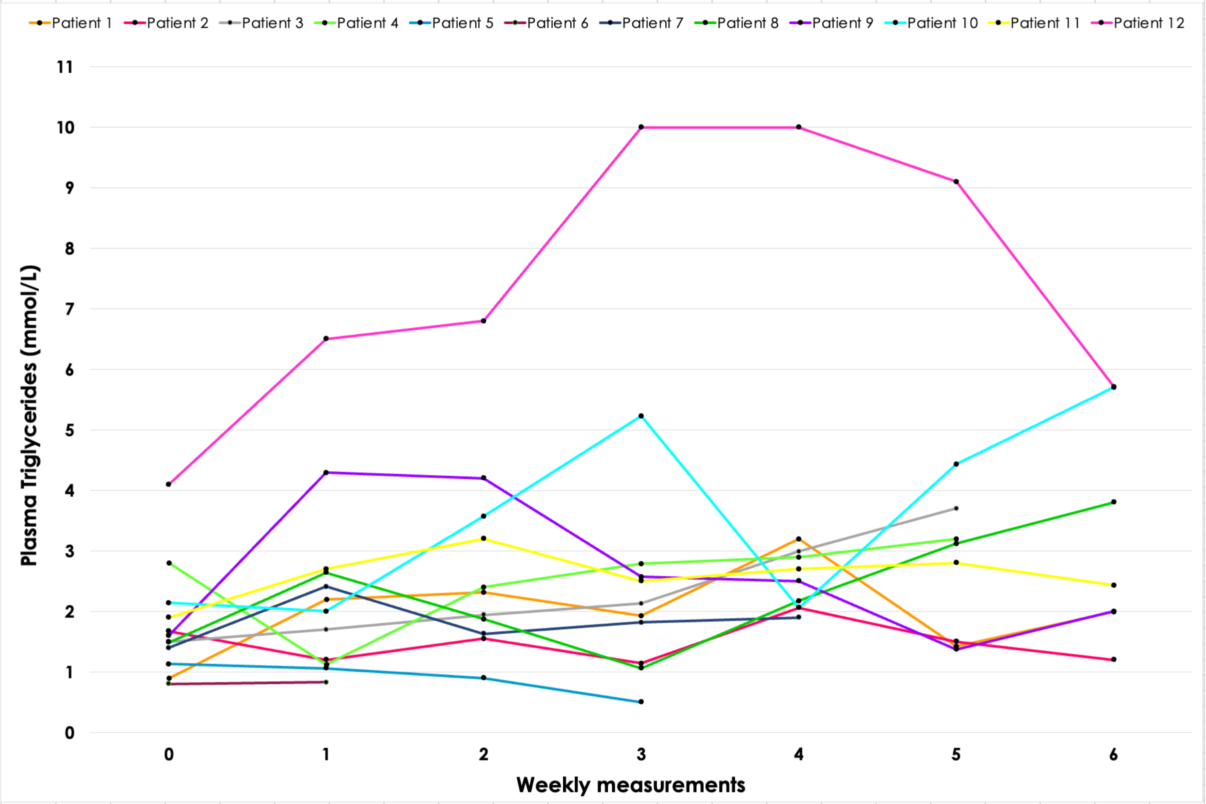

Figure 14 Weekly plasma triglycerides (mmol/L) measurements from baseline to end of week 6. Blood samples were taken postprandial after receiving a high fat meal and not in a fasted state. The high level of triglycerides in circulation after high fat meals could partly explain the elevated level of plasma triglycerides in some patients.

Supplementary Table 4 and 5. Changes in Body Weight and Body Composition

Table 4 Differences in weight between weighing at inclusion and completion/exclusion.

Weight difference between first and last measurement		
**Patient**	**Weight difference (kg)**	**Time between measurements**
1	+ 1.5	6 weeks
2	+ 0.8	5 weeks and 5 days
3	– 3.0	5 weeks and 4 days
4	+ 2.1	4 weeks and 2 days
5	+ 4,1	3 weeks
6	– 2.4	2 weeks and 2 days
7	– 0.1	4 weeks
8	+ 2.1	5 weeks
9	– 0.8	6 weeks
10	+ 0.8	6 weeks
11	– 0.7	5 weeks and 6 days
12	– 2.1	5 weeks and 5 days

Table 5 Differences in Body Weight, Body Mass Index, Fat Free Mass, Skeletal Muscle Mass, Body Fat, Body Fat Percentage, Total Body Water, Extracellular Body Water, and Intracellular Body Water, for all patients during the first two weeks of intervention and in nine patients during the first five weeks of intervention. Data is analysed with one-way repeated measures ANOVA.

**One-way repeated measures ANOVA**			
	df	***F***	***P-value***
**Body Weight**			
(n=9), Five first weeks	5	0.65	**0.66**
Error	40		
(n=12), Two first weeks	2	0.27	**0.77**
Error	22		
**BMI**			
(n=9), Five first weeks	5	0.78	**0.57**
Error	40		
(n=12), Two first weeks	2	0.38	**0.69**
Error	22		
**Fat Free Mass**			
(n=9), Five first weeks	5	1.32	**0.28**
Error	40		
(n=12), Two first weeks	2	0.91	**0.42**
Error	22		
**Skeletal Muscle Mass**			
(n=9), Five first weeks	5	0.82	**0.54**
Error	40		
(n=12), Two first weeks	2	0.92	**0.41**

Error	22		
**Body Fat**			
(n=9), Five first weeks	5	0.72	**0.61**
Error	40		
(n=12), Two first weeks	2	0.20	**0.82**
Error	22		
**Body Fat %**			
(n=9), Five first weeks	5	0.80	**0.56**
Error	40		
(n=12), Two first weeks	2	0.25	**0.78**
Error	22		
**Total Body Water**			
(n=9), Five first weeks	5	1.41	**0.24**
Error	40		
(n=12), Two first weeks	2	0.91	**0.42**
Error	22		
**Extracellular Water**			
(n=9), Five first weeks	5	2.00	**0.10**
Error	40		
(n=12), Two first weeks	2	0.77	**0.48**
Error	22		
**Intracellular Water**			
(n=9), Five first weeks	5	1.04	**0.41**
Error	40		
(n=12), Two first weeks	2	0.94	**0.40**
Error	22		

n, number; df, degrees of freedom

Supplementary Table 6. CONSORT 2010 checklist of information to include when reporting a pilot or feasibility trial

CONSORT 2010 checklist of information to include when reporting a pilot or feasibility trial*

Section/Topic	Item No	Checklist item	Reported on page No
**Title and abstract**			
	1a	Identification as a pilot or feasibility randomised trial in the title	1
	1b	Structured summary of pilot trial design, methods, results, and conclusions (for specific guidance see CONSORT abstract extension for pilot trials)	1
**Introduction**			
Background and objectives	2a	Scientific background and explanation of rationale for future definitive trial, and reasons for randomised pilot trial	2-3
	2b	Specific objectives or research questions for pilot trial	2-3
**Methods**			
Trial design	3a	Description of pilot trial design (such as parallel, factorial) including allocation ratio	3
	3b	Important changes to methods after pilot trial commencement (such as eligibility criteria), with reasons	3
Participants	4a	Eligibility criteria for participants	3
	4b	Settings and locations where the data were collected	3
	4c	How participants were identified and consented	3
Interventions	5	The interventions for each group with sufficient details to allow replication, including how and when they were actually administered	3
Outcomes	6a	Completely defined prespecified assessments or measurements to address each pilot trial objective specified in 2b, including how and when they were assessed	3-4
	6b	Any changes to pilot trial assessments or measurements after the pilot trial commenced, with reasons	na
	6c	If applicable, prespecified criteria used to judge whether, or how, to proceed with future definitive trial	4
Sample size	7a	Rationale for numbers in the pilot trial	4
	7b	When applicable, explanation of any interim analyses and stopping guidelines	na
Randomisation:			
Sequence generation	8a	Method used to generate the random allocation sequence	na
	8b	Type of randomisation(s); details of any restriction (such as blocking and block size)	na
Allocation concealment mechanism	9	Mechanism used to implement the random allocation sequence (such as sequentially numbered containers), describing any steps taken to conceal the sequence until interventions were assigned	na

Implementation	10	Who generated the random allocation sequence, who enrolled participants, and who assigned participants to interventions	na
Blinding	11a	If done, who was blinded after assignment to interventions (for example, participants, care providers, those assessing outcomes) and how	na
	11b	If relevant, description of the similarity of interventions	na
Statistical methods	12	Methods used to address each pilot trial objective whether qualitative or quantitative	4
**Results**			
Participant flow (a diagram is strongly recommended)	13a	For each group, the numbers of participants who were approached and/or assessed for eligibility, randomly assigned, received intended treatment, and were assessed for each objective	5-6
	13b	For each group, losses and exclusions after randomisation, together with reasons	5-6
Recruitment	14a	Dates defining the periods of recruitment and follow-up	6
	14b	Why the pilot trial ended or was stopped	na
Baseline data	15	A table showing baseline demographic and clinical characteristics for each group	7
Numbers analysed	16	For each objective, number of participants (denominator) included in each analysis. If relevant, these numbers should be by randomised group	6, 9, 12
Outcomes and estimation	17	For each objective, results including expressions of uncertainty (such as 95% confidence interval) for any estimates. If relevant, these results should be by randomised group	5-7
Ancillary analyses	18	Results of any other analyses performed that could be used to inform the future definitive trial	7
Harms	19	All important harms or unintended effects in each group (for specific guidance see CONSORT for harms)	5, 9
	19a	If relevant, other important unintended consequences	5, 9
**Discussion**			
Limitations	20	Pilot trial limitations, addressing sources of potential bias and remaining uncertainty about feasibility	8-13
Generalisability	21	Generalisability (applicability) of pilot trial methods and findings to future definitive trial and other studies	7-13
Interpretation	22	Interpretation consistent with pilot trial objectives and findings, balancing potential benefits and harms, and considering other relevant evidence	7-13
	22a	Implications for progression from pilot to future definitive trial, including any proposed amendments	13-14
**Other information**			
Registration	23	Registration number for pilot trial and name of trial registry	1, 3
Protocol	24	Where the pilot trial protocol can be accessed, if available	na
Funding	25	Sources of funding and other support (such as supply of drugs), role of funders	14
	26	Ethical approval or approval by research review committee, confirmed with reference number	3, 14

Citation: Eldridge SM, Chan CL, Campbell MJ, Bond CM, Hopewell S, Thabane L, et al. CONSORT 2010 statement: extension to randomised pilot and feasibility trials. BMJ. 2016;355.
*We strongly recommend reading this statement in conjunction with the CONSORT 2010, extension to randomised pilot and feasibility trials, Explanation and Elaboration for important clarifications on all the items. If relevant, we also recommend reading CONSORT extensions for cluster randomised trials, non-inferiority and equivalence trials, non-pharmacological treatments, herbal interventions, and pragmatic trials. Additional extensions are forthcoming: for those and for up to date references relevant to this checklist, see www.consort-statement.org.

Supplementary Table 7. TIDieR (Template for Intervention Description and Replication) Checklist

TIDieR
Template for Intervention Description and Replication

The TIDieR (Template for Intervention Description and Replication) Checklist*:
Information to include when describing an intervention and the location of the information

Item number	Item	Where located ** Primary paper (page or appendix number)	Other † (details)
	**BRIEF NAME**		
**1.**	Provide the name or a phrase that describes the intervention.	1	______________
	**WHY**		
**2.**	Describe any rationale, theory, or goal of the elements essential to the intervention.	2-3	______________
	**WHAT**		
**3.**	Materials: Describe any physical or informational materials used in the intervention, including those provided to participants or used in intervention delivery or in training of intervention providers. Provide information on where the materials can be accessed (e.g. online appendix, URL).	3	______________
**4.**	Procedures: Describe each of the procedures, activities, and/or processes used in the intervention, including any enabling or support activities.	3-4	______________
	**WHO PROVIDED**		
**5.**	For each category of intervention provider (e.g. psychologist, nursing assistant), describe their expertise, background and any specific training given.	na	______________
	**HOW**		
**6.**	Describe the modes of delivery (e.g. face-to-face or by some other mechanism, such as internet or telephone) of the intervention and whether it was provided individually or in a group.	3	______________
	**WHERE**		

**7.**	Describe the type(s) of location(s) where the intervention occurred, including any necessary infrastructure or relevant features.	3	______________
	**WHEN and HOW MUCH**		
**8.**	Describe the number of times the intervention was delivered and over what period of time including the number of sessions, their schedule, and their duration, intensity or dose.	3-4	______________
	**TAILORING**		
**9.**	If the intervention was planned to be personalised, titrated or adapted, then describe what, why, when, and how.	3-4	______________
	**MODIFICATIONS**		
**10.**[ǂ]	If the intervention was modified during the course of the study, describe the changes (what, why, when, and how).	5-7	______________
	**HOW WELL**		
**11.**	Planned: If intervention adherence or fidelity was assessed, describe how and by whom, and if any strategies were used to maintain or improve fidelity, describe them.	3-4	______________
**12.**[ǂ]	Actual: If intervention adherence or fidelity was assessed, describe the extent to which the intervention was delivered as planned.	5-7	______________

** **Authors -** use N/A if an item is not applicable for the intervention being described. **Reviewers** – use '?' if information about the element is not reported/not sufficiently reported.

† If the information is not provided in the primary paper, give details of where this information is available. This may include locations such as a published protocol or other published papers (provide citation details) or a website (provide the URL).
ǂ If completing the TIDieR checklist for a protocol, these items are not relevant to the protocol and cannot be described until the study is complete.

* We strongly recommend using this checklist in conjunction with the TIDieR guide (see *BMJ* 2014;348:g1687) which contains an explanation and elaboration for each item.
* The focus of TIDieR is on reporting details of the intervention elements (and where relevant, comparison elements) of a study. Other elements and methodological features of studies are covered by other reporting statements and checklists and have not been duplicated as part of the TIDieR checklist. When a **randomised trial** is being reported, the TIDieR checklist should be used in conjunction with the CONSORT statement (see www.consort-statement.org) as an extension of **Item 5 of the CONSORT 2010 Statement.** When a **clinical trial protocol** is being reported, the TIDieR checklist should be used in conjunction with the SPIRIT statement as an extension of **Item 11 of the SPIRIT 2013 Statement** (see www.spirit-statement.org). For alternate study designs, TIDieR can be used in conjunction with the appropriate checklist for that study design (see www.equator-network.org)
